# Synthesis and Smo Activity of Some Novel Benzamide Derivatives

**DOI:** 10.3390/molecules23010085

**Published:** 2017-12-31

**Authors:** Huaiwei Ding, Kai Chen, Bingke Song, Chenglong Deng, Wei Li, Li Niu, Mengxuan Bai, Hongrui Song, Lijuan Zhang

**Affiliations:** Key Laboratory of Structure-Based Drug Design and Discovery, Ministry of Education, Shenyang Pharmaceutical University, Shenyang 110016, China; dinghuaiwei627@163.com (H.D.); kylechen0322@163.com (K.C.); s13050245586@163.com (B.S.); m15694145159@163.com (C.D.); 15757196069@163.com (W.L.); 18435154073@163.com (L.N.); baimengxuan_2015@126.com (M.B.)

**Keywords:** benzamide, hedgehog signaling, Gli-luc luciferase activity

## Abstract

Two series of benzamides compounds bearing piperidine groups were synthesized and the Gli-luc luciferase activity was screened by Gys-luc luciferase gene detection method. Compound **5q** showed promising inhibition of hedgehog (Hh) signaling pathway. To further verify whether the Hh inhibitory activities of the target compounds are derived from their inhibition to the Smoothened (Smo) receptor, the compounds with good potency were evaluated in a fluorescence competitive displacement assays, the results showed the Smo inhibitory potency of these compounds correlated well with their Hh inhibition, which suggested that the observed Hh activity was driven by Smo inhibitors.

## 1. Introduction

Hedgehog (Hh) signaling pathway is crucial for the development of various embryonic tissues in invertebrate and vertebrate development, including brain, spinal cord, lungs, gut, and hematopoietic cells [1]. Abnormal mutations of the Hh signaling pathway can lead to cell proliferation and tumor growth, especially in basal cell carcinoma and medulloblastoma [2]. Recent studies have shown that activation of the Hh signaling pathway not only increases the risk of cancer patients, but also plays an important role in reducing the efficacy and tolerability of drugs [3,4]. At present, the inhibitor of Hh signaling pathway is mainly composed of the direct action of Smo [5,6,7], acting on the upstream of Smo [8], acting on the downstream of Smo [9], and other inhibitors, which are not very clear [10,11]. 

**Figure 1:**
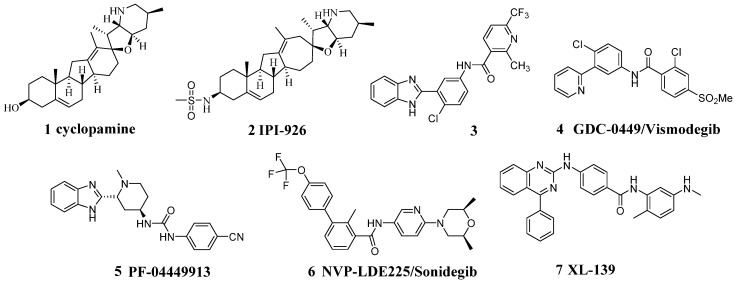
Promising Smo inhibitors.

Cyclopamine (**1**, Figure 1) is considered to be the most studied small molecule Hh signaling pathway inhibitor in natural products, and has been shown to be the first inhibitor of Smo receptors [12]. There are some reported compounds which have been developed that have a good inhibitory effect on Smo, such as IPI-926 (**2**) [13] and compound (**3**) [14], and GDC-0449 (**4**), which exhibited good pharmacokinetic and physicochemical properties and became a valuable Smo inhibitor; it was approved by the Food and Drug Administration (FDA) in 2012 under the trade name Vismodegib [6]. The benzimidazole-based compound PF-04449913 (**5**), which was developed by Pfizer, is currently undergoing clinical trials [15]. At the same time, NVP-LDE225 (**6**) is a promising inhibitor which was approved by the FDA in 2015, with the product name Sonidegib [16]. Compound (**7**) was developed by Exelixis and Squibb as a Hh pathway inhibitor and it is undergoing clinical phase II trials at present [17].

**Figure 2:**
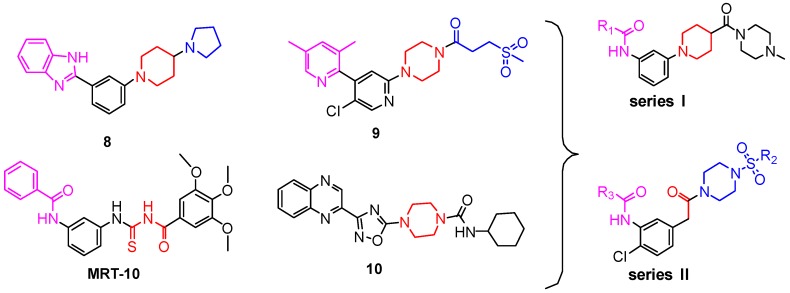
The design strategy of benzamide target compounds.

Through the analysis of listed drug (Vismodegib and Sonidegib), and MRT-10 [18], we found that the basic skeleton is a meta-disubstituted aromatic ring, one end of which is an amide (or an inverted amide) bond, the other end being an aromatic ring structure. In the design of the target compounds, we first introduced the left-side benzamide fragment of compound MRT-10, which is also a bioisostere of the benzimidazole group in compound (**8**). Then we introduced different electron-withdrawing groups or electron-donating groups into the benzene ring, to change the electrical properties of the molecules. On the other side of the structure, the results of the previous work [19] showed that the amide structure combined with the Smo receptor through the hydrogen bond, so the amide structure might be a necessary group for activity. Considering that the compounds (**8**) and (**9**) are based on more rigid piperidine or piperazine to obtain better inhibition activity of Hh, we used a rigid piperidine ring as a linker at the other end of the structural design, and then adopted water-soluble formyl piperazine at the right side of the piperidine, which aims to reduce lipo-hydro partition coefficient of the target molecules (Figure 2). On the other hand, we replaced the pyridine ring in Vismodegib with the arylcarboxamide group and the chlorophenyl group remained unchanged; the carbon atom was substituted for the nitrogen atom of the amide bond; the sulfonamide piperazinyl group was substituted for 2-chloro-4-methanesulfophenyl. In summary, we designed and synthesized the new benzamide compounds **5a**–**q** and **8a**–**c** which contain a piperidine group, and their biological activities were evaluated.

## 2. Results and Discussion

### 2.1. Chemistry

**Scheme 1:**
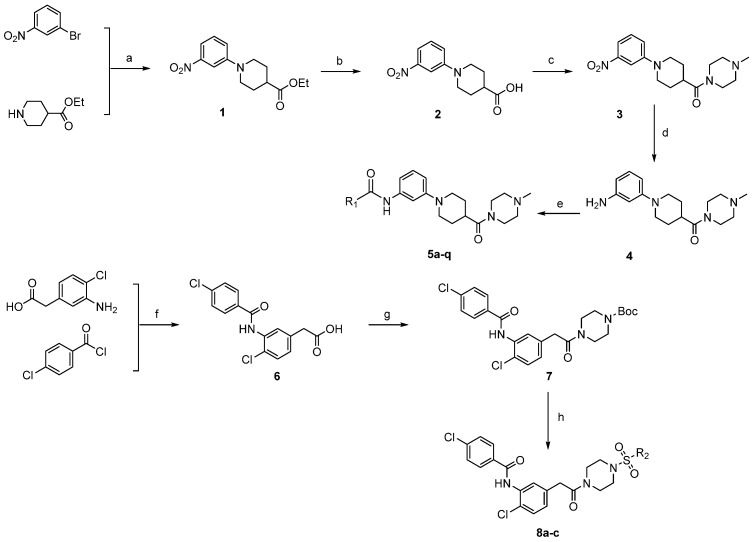
Synthetic procedures of compounds **1**–**4**, **6**–**7**, **5a**–**q** and **8a**–**c**. *Reagents and conditions:* (a) Pd(OAc)_2_, BINAP, toluene, 100 °C, 12 h; (b) NaOH, 1,4-dioxane, 70 °C; (c) DIEA, HOBt, EDCI, tetrahydrofuran, *N*-methylpiperazine, 50 °C, 2 h; (d) Zn/AcOH, EtOH, 80 °C,12 h; (e) HATU, CH_2_Cl_2_, ethyl acetate, room temperature (rt), overnight; (f) Et_3_N, dichloromethane, 0 °C, 2 h; (g) 1-boc-piperazine, DIEA, HOBt, EDCI, rt, 24h; (h) і, TFA: CH_2_Cl_2_ = 50%, rt, 4 h; іі, sulfonyl chloride, Et_3_N, CH_2_Cl_2_, 0 °C, 12 h.

All the agents, unless mentioned otherwise, are commercially available and were directly used without further purification. The synthesis route of target compounds was shown in Scheme 1. Nitrobromobenzene reacted with piperidine-4-carboxylic acid ethyl ester to obtain intermediate **1**, which was further hydrolyzed and acidized to get intermediate **2**, and then intermediate **2** reacted with *N*-methylpiperazine to obtain intermediate **3**. Compound **3** was reduced to obtain intermediate **4**. The final compounds **5a**–**q** were formed by reacting with benzoic acid compounds with different groups through amide condensation reaction. Compounds **8a**–**c** were obtained by three different acylation reactions. The structures of the new analogs were characterized by Proton Nuclear Magnetic Resonance Spectra (^1^H-NMR) and Mass Spectra (MS). The detailed synthesis methods and characterizations are presented in Supplementary Materials.

### 2.2. In Vitro Bioactivity

In the Hh signaling pathway activity test, we used the Gli-Luc double luciferase detection system, which is widely used to test the inhibitory activity of the Hh signaling pathway. In the experiment, we used MRT-10 as the lead compound, and the Vismodegib as the positive control drug. The results of the specific tests were shown in Table 1.

In NIH3T3 cells transfected with Gli luciferase experiments, we found that compounds with substituents on the benzene ring (other than nitro substituents) had better inhibitory activity against Hh signaling pathways than substituents without substituents, such as compounds **5a** (IC_50_ = 28.52 μM) and **5b** (IC_50_ = 12.47 μM). In the comparison of the activity of the analogs substituted with different halogens, it was found that the activity of the bromide was better than that of the chloride and fluorinated compounds, but the activity of halogen substitutes was two to three times better than that of alkyl substitutes. The activity of the para-substituted halogen compound was slightly better than that of the meta-substituted compound, for example the activity of the compound **5b** was better than that of the compound **5c** (IC_50_ = 14.63 μM). Compound **5j** (IC_50_ = 4.74 μM) was more potent than compound **5h** (IC_50_ = 7.85 μM), **5b** and **5d** (IC_50_ = 15.51 μM), suggesting that electron-donating group can improve the activity. When the benzene was replaced with pyridine or sulfonyl group, the activity of the compound was improved. Among them, compound **5q** showed the best activity with an IC_50_ value of 1.26 μM.

In order to develop more core structure, we synthesized another series compounds **8a** to **8c**, but the activities of this series were not as good as that of the first series.

To determine whether the Hh inhibitory activities of the target compounds were derived from their inhibition to the Smo receptor, a binding assay was used. As shown in Table 2, several compounds with better activity were subjected to Smo protein binding experiments. It was found that these compounds inhibited the binding of boron dipyrromethene (BODIPY)-labelled cyclopamine to Smo protein in HEK293 cells in different degrees. The results showed that the activity of these compounds was positively correlated with Gli-luc data, suggesting that these compounds inhibited the Hh signaling activity by interacting with Smo receptors. Although the activity test showed that the activity of 20 target compounds had a certain gap with the control Vismodegib, the experimental data of these compounds laid the foundation for further structural optimization in the later stage.

### 2.3. Molecular Docking and Simulation

**Figure 3:**
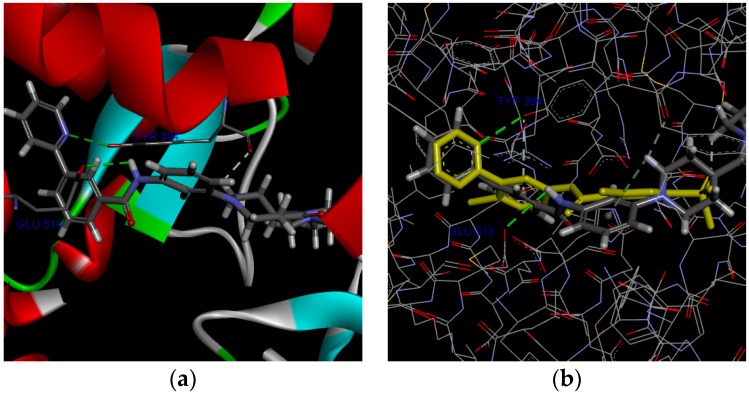
(**a**) Key interactions of **5q** in the active site of Smo. (**b**) The binding pose of **5q** and Vismodegib in the active site of Smo. Vismodegib was highlighted with yellow. Atoms are color-coded: Grey, carbon; white, hydrogen; red, oxygen; purple, nitrogen, bonds are color-coded: Green, classical hydrogen bonds; white, non-classical hydrogen bonds.

Molecular docking study was performed to elucidate the binding model of **5q** in the binding site of Smo with molecular docking software Discovery Studio 3.0. Compared with the control drug Sonidegib. As shown in Figure 3a, **5q** forms hydrogen bonds with amino acid Tyr394 and amino acid Glu518. As shown in Figure 3b, the folded form of compound **5q** in the protein pocket was very similar to that of Vismodegib. These formation of hydrogen bonds suggested that **5q** can exactly interact with the catalytic domain of Smo and also indicated that **5q** might be a potent Smo inhibitor (PDB entry code: 5L7I).

## 3. Experimental

### 3.1. Materials and Reagents

All reagents and solvents were commercially available without further purification. ^1^H-NMR spectra were recorded on 400 Bruker NMR spectrometer (Rheinstetten, Germany) with tetramethylsilane (TMS) as an internal standard. All chemical shifts are reported in ppm (δ) and coupling constants (*J*) are in hertz (Hz). Mass measurements were performed using electrospray ionization. Follow-up of the reactions and checking the homogeneity of the compounds were made by thin layer chromatography (TLC) on silica gel-protected glass plates and the spots were detected by exposure to an ultra-violet (UV)-lamp at λ254 and λ365.

### 3.2. Chemical Synthesis

#### 3.2.1. Procedure for Preparation Intermediates of **1**–**4**

*Ethyl 1-(3-nitrophenyl)piperidine-4-carboxylate* (**1**): Palladium acetate (0.157 g, 0.0007 mol), (±)-2,2′-Bis(diphenylphosphino)-1,1′-binaphthalene (BINAP, 0.64 g, 0.001 mol) were dissolved in 10 mL of toluene and stirred for 30 min under nitrogen at room temperature. Ethyl piperidine-4-carboxylate (3.45 g, 0.022 mol) was added slowly to the reaction solution, stirred for 5 min. Then, 1-bromo-3-nitrobenzene (5.3 g, 0.026 mol), cesium carbonate (19.5 g, 0.06 mol) were added. The reaction solution was stirred under a nitrogen atmosphere at 100 °C for 12 h. After the reaction was completed, the reaction solution was cooled to room temperature, filtered, then the insoluble material was removed, the filtrate was washed with saturated brine (20 mL × 3) and the organic phase was evaporated to dryness and the product was purified by column chromatography on silica gel (ethyl acetate: *n*-hexane = 10:1) to get **1** (4.3 g, 71% yield). ^1^H-NMR (400 MHz, DMSO-*d*_6_) δ 7.65–7.34 (m, 4H, Ar-H), 4.13–4.03 (m, 2H, CH_2_), 3.82–3.72 (m, 2H, piperidine), 2.96–2.84 (m, 2H, piperidine), 2.59–2.47 (m, 1H, piperidine), 1.92 (dd, *J* = 13.7, 3.7 Hz, 2H, piperidine), 1.76–1.54 (m, 2H, piperidine), 1.19 (t, *J* = 7.1 Hz, 3H, CH_3_). ESI-MS *m*/*z*: 278.9 [M + H]^+^. 

*1-(3-Nitrophenyl)piperidine-4-carboxylic acid* (**2**): **1** (3 g, 0.01 mol) was dissolved in 20 mL of dioxane, then sodium hydroxide solution (1.3 g of sodium hydroxide, 30 mL of deionized water) was added and the reaction was slowly warmed to 70 °C and incubated for 3 h. The reaction solution was cooled to room temperature, and the solvent was distilled off under reduced pressure. 10 mL of deionized water was added, and the pH of the aqueous layer was adjusted to 4.7 with 1 mol/L hydrochloric acid. It was slowly cooled to 0 °C, stirred for another 3 h, and filtered to give a slightly yellow solid which was washed with deionized water (20 mL × 2) and dried under reduced pressure to give product **2** (2.7 g, 90% yield). ^1^H-NMR (400 MHz, DMSO-*d*_6_) δ 12.26 (s, 1H, COOH), 7.69–7.36 (m, 4H, Ar-H), 3.82–3.72 (m, 2H, piperidine), 2.96–2.84 (m, 2H, piperidine), 2.48–2.35 (m, 1H, piperidine), 1.92 (dd, *J* = 13.6, 3.9 Hz, 2H, piperidine), 1.70–1.55 (m, 2H, piperidine). ESI-MS *m*/*z*: 251.0 [M + H]^+^.

*(4-Methylpiperazin-1-yl)(1-(3-nitrophenyl)piperidin-4-yl)methanone* (**3**): *N*-Methylpiperazine (1.2 g, 0.012 mol), *N*-Ethyldiisopropylamine (DIPEA, 4.16 mL, 0.036 mol) were dissolved in 20 mL of tetrahydrofuran. **2** (2.0 g, 0.012 mol), *N*-hydroxybenzotrizole (HOBT, 1.8 g, 0.012 mol) and 1-Ethyl-3-(3-dimethylaminopropyl)carbodiimide hydrochloride (EDCI, 1.54 g, 0.012 mol) were added with stirring. After 24 h of reaction, the reaction was complete. The reaction solution was poured into water and extracted with methylene chloride. The reaction mixture was dried, then it was purified by column chromatography on silica gel (dichloromethane:methanol = 30:1) to give product **3** (2.4 g, 61% yield). ^1^H-NMR (400 MHz, DMSO-*d*_6_) δ 7.86–7.42 (m, 4H, Ar-H), 4.33 (dd, *J* = 88.1, 14.4 Hz, 2H, piperazine), 3.95–3.73 (m, 2H, piperidine), 3.68–3.28 (m, 3H, piperidine and piperazine), 3.14–2.94 (m, 5H, piperidine and piperazine), 2.95–2.86 (m, H, piperidine), 2.74 (d, *J* = 4.6 Hz, 3H, CH_3_), 1.80–1.61 (m, 4H, piperidine). ESI-MS *m*/*z*: 333.1 [M + H]^+^.

*(1-(3-Aminophenyl)piperidin-4-yl)(4-methylpiperazin-1-yl)methanone* (**4**): **3** (0.12 g, 0.00036 mol) was dissolved in 10 mL ethanol, and ammonium chloride (0.1 g, 0.0018 mol), zinc powder (0.118 g, 0.0018 mol) and 2 mL glacial acetic acid were added and reacted at 80 °C for 12 h. The reaction mixture was then filtered and evaporated to dryness, adjusted the aqueous layer to pH 10 with saturated sodium carbonate, solid was filtered off, then the filtrate was extracted with ethyl acetate for three times. The combined organic layers were evaporated to dryness. The product was purified by column chromatography on silica gel (ethyl acetate:*n*-hexane = 20:1) to give product **4** (0.094 g, 86% yield). ^1^H-NMR (400 MHz, DMSO-*d*_6_) δ 6.84 (t, *J* = 8.0 Hz, 1H, Ar-H), 6.17 (t, *J* = 2.2 Hz, 1H, Ar-H), 6.13 (dd, *J* = 8.1, 2.3 Hz, 1H, Ar-H), 6.02 (dd, *J* = 7.8, 1.9 Hz, 1H, Ar-H), 3.72 (s, 2H, NH_2_), 3.65–3.54 (m, 4H, piperazine), 3.11–3.03 (m, 1H, piperidine), 2.94–2.63 (m, 8H, piperidine and piperazine), 2.55 (s, 3H, CH_3_), 1.69–1.62 (m, 4H, piperidine). ESI-MS *m*/*z*: 303.3 [M + H]^+^.

#### 3.2.2. General Procedure for Preparation of Target Compounds of **5a**–**q**

**4** (0.1 g, 0.0002 mol), 1-[Bis(dimethylamino)methylene]-1*H*-1,2,3-triazolo[4,5-b]pyridinium 3-oxid hexafluorophosphate (HATU, 0.2 g, 0.0004 mol), and substituted benzoic acid (0.0003 mol) were dissolved in 2 mL of dichloromethane followed by DIPEA (1 g, 0.7 mmol), the reaction was allowed to react at room temperature for 20 h, 20 mL of methylene chloride was added, and the reaction solution was washed with saturated sodium bicarbonate solution (10 mL × 2). The reaction solution was dried over anhydrous sodium sulfate and evaporated to dryness under reduced pressure to give a yellow oil which was purified by column chromatography on silica gel (*n*-hexane:ethyl acetate = 10:1) to get product **5a**–**q**.

Compounds **5a**–**q** was synthesized according to the above steps. The spectral data are as follows:

*N-(3-(4-(4-Methylpiperazine-1-carbonyl)piperidin-1-yl)phenyl)benzamide* (**5a**): yield 63%. ^1^H-NMR (400 MHz, CDCl_3_) δ 7.90–7.79 (m, 3H, Ar-H and NH), 7.59–7.44 (m, 4H, Ar-H), 7.22 (t, *J* = 16.2 Hz, 1H, Ar-H), 6.94 (d, *J* = 8.8 Hz, 1H, Ar-H), 6.73 (dd, *J* = 8.3, 1.9 Hz, 1H, Ar-H), 3.79 (d, *J* = 12.4 Hz, 2H, piperazine), 3.65 (s, 2H, piperazine), 3.55 (s, 2H, piperidine), 2.84–2.73 (m, 2H, piperidine), 2.61 (tt, *J* = 11.3, 3.5 Hz, 1H, piperidine), 2.40 (d, *J* = 16.9 Hz, 4H, piperazine), 2.32 (s, 3H, CH_3_), 2.08–1.90 (m, 2H, piperidine), 1.82–1.79 (m, *J* = 12.7 Hz, 2H, piperidine). ESI-MS *m*/*z*: 407.1 [M + H]^+^.

*4-Chloro-N-(3-(4-(4-methylpiperazine-1-carbonyl)piperidin-1-yl)phenyl)benzamide* (**5b**): yield 61%. ^1^H-NMR (400 MHz, CDCl_3_) δ 7.87–7.78 (m, 3H, Ar-H and NH), 7.49–7.37 (m, Ar-H), 7.23 (d, *J* = 8.1 Hz, 1H, Ar-H), 6.93 (d, *J* = 7.9 Hz, 1H, Ar-H), 6.76–6.71 (m, 1H, Ar-H), 3.77 (d, *J* = 12.4 Hz, 2H, piperazine), 3.68–3.52 (m, 4H, piperazine and piperidine), 2.83–2.73 (m, 2H, piperidine), 2.61 (ddd, *J* = 11.3, 7.1, 3.5 Hz, 1H, piperidine), 2.40 (d, *J* = 18.5 Hz, 4H, piperazine), 2.32 (s, 3H, CH_3_), 1.96 (tt, *J* = 12.2, 6.6 Hz, piperidine), 1.76 (d, *J* = 17.8 Hz, 2H, piperidine). ESI-MS *m*/*z*: 441 [M + H]^+^.

*3-Chloro-N-(3-(4-(4-methylpiperazine-1-carbonyl)piperidin-1-yl)phenyl)benzamide* (**5c**): yield 65%. ^1^H-NMR (400 MHz, CDCl_3_) δ 7.89–7.80 (m, 2H, Ar-H and NH), 7.74 (d, *J* = 7.6 Hz, 1H, Ar-H), 7.51 (d, *J* = 7.7 Hz, 1H, Ar-H), 7.42 (t, *J* = 7.6 Hz, 2H, Ar-H), 7.22 (t, *J* = 8.1 Hz, 1H, Ar-H), 6.96 (d, *J* = 7.7 Hz, 1H, Ar-H), 6.74 (d, *J* = 8.2 Hz, 1H, Ar-H), 3.78 (d, *J* = 12.3 Hz, 2H, piperazine), 3.69–3.62 (m, 2H, piperazine), 3.56 (s, 2H, piperidine), 2.83–2.74 (m, 2H, piperidine), 2.64–2.56 (m, 1H, piperidine), 2.42 (d, *J* = 18.6 Hz, 4H, piperazine), 2.33 (s, 3H, CH_3_), 2.01–1.95 (m, 2H, piperidine), 1.84–1.76 (m, 2H, piperidine). ESI-MS *m*/*z*: 441.1 [M + H]^+^.

*4-Fluoro-N-(3-(4-(4-methylpiperazine-1-carbonyl)piperidin-1-yl)phenyl)benzamide* (**5d**): yield 55%. ^1^H-NMR (400 MHz, CDCl_3_) δ 7.91–7.85 (m, 2H, Ar-H), 7.78 (s, 1H, NH), 7.43 (s, 1H, Ar-H), 7.24–7.14 (m, 2H, Ar-H), 6.93 (d, *J* = 7.9 Hz, 1H, Ar-H), 6.75–6.71 (m, 1H, Ar-H), 3.78 (d, *J* = 12.4 Hz, 2H, piperazine), 3.68–3.53 (m, 4H, piperazine and piperidine), 2.83–2.75 (m, 2H, piperidine), 2.65–2.57 (m, 1H, piperidine), 2.41 (d, *J* = 17.1 Hz, 4H, piperazine), 2.32 (s, 3H, CH_3_), 2.01–1.93 (m, 2H, piperidine), 1.80 (d, *J* = 12.6 Hz, 2H, piperidine). ESI-MS *m*/*z*: 425.1 [M + H]^+^.

*3-Fluoro-N-(3-(4-(4-methylpiperazine-1-carbonyl)piperidin-1-yl)phenyl)benzamide* (**5e**): yield 51%. ^1^H-NMR (400 MHz, CDCl_3_) δ 7.83 (s, 1H, NH), 7.64–7.56 (m, 2H, Ar-H), 7.45 (d, *J* = 9.4 Hz, 2H, Ar-H), 7.25–7.18 (m, 2H, Ar-H), 6.94 (d, *J* = 7.8 Hz, 1H, Ar-H), 6.74 (d, *J* = 8.3 Hz, 1H, Ar-H), 3.78 (d, *J* = 12.4 Hz, 2H, piperazine), 3.65 (s, 2H, piperazine), 3.55 (s, 2H, piperidine), 2.79 (t, *J* = 11.6 Hz, 2H, piperidine), 2.61 (t, *J* = 11.3 Hz, 1H, piperidine), 2.41 (d, *J* = 18.0 Hz, 4H, piperazine), 2.32 (s, 3H, CH_3_), 2.02–1.92 (m, 2H, piperidine), 1.80 (d, *J* = 12.0 Hz, 2H, piperidine). ESI-MS *m*/*z*: 425.1 [M + H]^+^.

*N-(3-(4-(4-methylpiperazine-1-carbonyl)piperidin-1-yl)phenyl)-3-nitrobenzamide* (**5f**): yield 32%. ^1^H-NMR (400 MHz, CDCl_3_) δ 8.72 (s, 1H, NH), 8.41–8.32 (m, 2H, Ar-H), 8.26 (d, *J* = 7.7 Hz, 1H, Ar-H), 7.67 (t, *J* = 8.0 Hz, 1H, Ar-H), 7.39 (s, 1H, Ar-H), 7.22 (t, *J* = 8.1 Hz, 1H, Ar-H), 7.04 (d, *J* = 7.7 Hz, 1H, Ar-H), 6.76–6.71 (m, 1H, Ar-H), 3.76 (d, *J* = 12.5 Hz, 2H, piperazine), 3.67–3.53 (m, 4H, piperazine and piperidine), 2.83–2.74 (m, 2H, piperidine), 2.62 (tt, *J* = 11.2, 3.4 Hz, 1H, piperidine), 2.39 (d, *J* = 27.0 Hz, 4H, piperazine), 2.31 (s, 3H, CH_3_), 2.00–1.89 (m, 2H, piperidine), 1.78 (d, *J* = 19.8 Hz, 2H, piperidine). ESI-MS *m*/*z*: 452.1 [M + H]^+^.

*N-(3-(4-(4-methylpiperazine-1-carbonyl)piperidin-1-yl)phenyl)-4-nitrobenzamide* (**5g**): yield 31%. ^1^H-NMR (400 MHz, CDCl_3_) δ 8.49 (s, 1H, NH), 8.29–8.24 (m, 2H, Ar-H), 8.07–8.02 (m, 2H, Ar-H), 7.37 (s, 1H, Ar-H), 7.20 (t, *J* = 8.1 Hz, 1H, Ar-H), 7.01 (d, *J* = 7.7 Hz, 1H, Ar-H), 6.75–6.68 (m, 1H, Ar-H), 3.74 (d, *J* = 12.4 Hz, 2H, piperazine), 3.63–3.52 (m, 4H, piperazine and piperidine), 2.81–2.73 (m, 2H, piperidine), 2.65–2.58 (m, 1H, piperidine), 2.41 (d, *J* = 27.1 Hz, 4H, piperazine), 2.32 (s, 3H, CH_3_), 1.91 (tt, *J* = 12.3, 6.7 Hz, 2H, piperidine), 1.76 (d, *J* = 12.3 Hz, 2H, piperidine). ESI-MS *m*/*z*: 452.1 [M + H]^+^.

*4-Bromo-N-(3-(4-(4-methylpiperazine-1-carbonyl)piperidin-1-yl)phenyl)benzamide* (**5h**): yield 65%. ^1^H-NMR (400 MHz, CDCl_3_) δ 7.78 (s, 1H, NH), 7.76–7.71 (m, 2H, Ar-H), 7.65–7.59 (m, 2H, Ar-H), 7.43 (s, 1H, Ar-H), 7.22 (t, *J* = 8.1 Hz, 1H, Ar-H), 6.93 (d, *J* = 7.8 Hz, 1H, Ar-H), 6.76–6.71 (m, 1H, Ar-H), 3.78 (d, *J* = 12.5 Hz, 2H, piperazine), 3.67–3.53 (m, 4H, piperazine and piperidine), 2.82–2.74 (m, 2H, piperidine), 2.64–2.57 (m, 1H, piperidine), 2.41 (d, *J* = 16.3 Hz, 4H, piperazine), 2.33 (s, 3H, CH_3_), 2.01–1.92 (m, 2H, piperidine), 1.80 (d, *J* = 12.6 Hz, 2H, piperidine). ESI-MS *m*/*z*: 487.0 [M + H]^+^.

*3-Methyl-N-(3-(4-(4-methylpiperazine-1-carbonyl)piperidin-1-yl)phenyl)benzamide* (**5i**): yield 75%. ^1^H-NMR (400 MHz, CDCl_3_) δ 7.77 (s, 1H, NH), 7.68 (s, 1H, Ar-H), 7.66–7.61 (m, 1H, Ar-H), 7.49 (s, 1H, Ar-H), 7.38–7.33 (m, 2H, Ar-H), 7.22 (t, *J* = 8.1 Hz, 1H, Ar-H), 6.96–6.92 (m, 1H, Ar-H), 6.73 (dd, *J* = 8.3, 1.8 Hz, 1H, Ar-H), 3.79 (d, *J* = 12.4 Hz, 2H, piperazine), 3.71–3.56 (m, 4H, piperazine and piperidine), 2.82–2.75 (m, 2H, piperidine), 2.65–2.57 (m, 1H, piperidine), 2.45 (d, *J* = 12.9 Hz, 7H, piperazine and CH_3_), 2.35 (s, 3H, CH_3_), 2.01–1.93 (m, 2H, piperidine), 1.81 (d, *J* = 12.3 Hz, 2H, piperidine). ESI-MS *m*/*z*: 421.1 [M + H]^+^.

*3-Methoxy-N-(3-(4-(4-methylpiperazine-1-carbonyl)piperidin-1-yl)phenyl)benzamide* (**5j**): yield 47%. ^1^H-NMR (400 MHz, CDCl_3_) δ 7.86–7.82 (m, 2H, Ar-H and NH), 7.76 (s, 1H, Ar-H), 7.48 (s, 1H, Ar-H), 7.21 (t, *J* = 8.1 Hz, 1H, Ar-H), 6.99–6.95 (m, 2H, Ar-H), 6.92 (d, *J* = 7.8 Hz, 1H, Ar-H), 6.74–6.70 (m, 1H, Ar-H), 3.87 (s, 3H, OCH_3_), 3.78 (d, *J* = 12.4 Hz, 2H, piperazine), 3.68–3.56 (m, 4H, piperazine and piperidine), 2.81–2.74 (m, 2H, piperidine), 2.62–2.57 (m, 1H, piperidine), 2.45 (d, *J* = 14.5 Hz, 4H, piperazine), 2.35 (s, 3H, CH_3_), 2.01–1.95 (m, 2H, piperidine), 1.80 (d, *J* = 12.7 Hz, 2H, piperidine). ESI-MS *m*/*z*: 459.1 [M + Na]^+^.

*2-Methyl-N-(3-(4-(4-methylpiperazine-1-carbonyl)piperidin-1-yl)phenyl)benzamide* (**5k**): yield 47%. ^1^H-NMR (400 MHz, CDCl_3_) δ 7.52–7.42 (m, 3H, Ar-H and NH), 7.36 (t, *J* = 7.4 Hz, 1H, Ar-H), 7.26–7.16 (m, 3H, Ar-H), 6.91 (d, *J* = 7.1 Hz, 1H, Ar-H), 6.73 (d, *J* = 7.9 Hz, 1H, Ar-H), 3.78 (d, *J* = 11.6 Hz, 2H, piperazine), 3.70–3.57 (m, 4H, piperazine and piperidine), 2.79 (t, *J* = 12.0 Hz, 2H, piperidine), 2.60 (t, *J* = 11.3 Hz, 1H, piperidine), 2.52–2.43 (m, 7H, piperazine and CH_3_), 2.36 (s, 3H, CH_3_), 1.97 (d, *J* = 11.1 Hz, 2H, piperidine), 1.80 (d, *J* = 12.5 Hz, 2H, piperidine). ESI-MS *m*/*z*: 443.1 [M + H]^+^.

*3*,*5-Dimethoxy-N-(3-(4-(4-methylpiperazine-1-carbonyl)piperidin-1-yl)phenyl)benzamide* (**5l**): yield 61%. ^1^H-NMR (400 MHz, CDCl_3_) δ 7.80 (s, 1H, NH), 7.49 (s, 1H, Ar-H), 7.20 (s, 1H, Ar-H), 7.00–6.96 (m, 2H, Ar-H), 6.96 (m, 2H, Ar-H), 6.95–6.90 (m, 1H, Ar-H), 6.95–6.90 (m, 1H, Ar-H), 6.75–6.71 (m, 1H, Ar-H), 6.63–6.60 (m, 1H, Ar-H), 3.85 (s, 6H, OCH_3_), 3.81–3.77 (m, 2H, piperazine), 3.74–3.61 (m, 4H, piperazine and piperidine), 2.81–2.76 (m, 2H, piperidine), 2.62 (d, *J* = 11.2 Hz, piperidine), 2.57–2.49 (m, 4H, piperazine), 2.40 (s, 3H, CH_3_), 2.00–1.94 (m, 2H, piperidine), 1.82 (d, *J* = 12.7 Hz, 2H, piperidine). ESI-MS *m*/*z*: 467.2 [M + H]^+^.

*2-Bromo-N-(3-(4-(4-methylpiperazine-1-carbonyl)piperidin-1-yl)phenyl)benzamide* (**5m**): yield 42%. ^1^H-NMR (400 MHz, CDCl_3_) δ 7.69 (s, 1H, NH), 7.65–7.61 (m, Ar-H), 7.45 (s, 1H, NH), 7.41 (d, *J* = 7.4 Hz, 1H, Ar-H), 7.34–7.29 (m, 1H, Ar-H), 7.22 (t, *J* = 8.1 Hz, 1H, Ar-H), 6.94 (d, *J* = 7.8 Hz, 1H, Ar-H), 6.76–6.72 (m, 1H, Ar-H), 3.78 (d, *J* = 12.4 Hz, 2H, piperazine), 3.70–3.55 (m, 4H, piperazine and piperidine), 2.79 (t, *J* = 11.8 Hz, 2H, piperidine), 2.64–2.57 (m, 1H, piperidine), 2.44 (d, *J* = 15.2 Hz, 4H, piperazine), 2.34 (s, 3H, CH_3_), 2.01–1.94 (m, 2H, piperidine), 1.82 (d, *J* = 12.6 Hz, 2H, piperidine). ESI-MS *m*/*z*: 487.1 [M + H]^+^.

*N-(3-(4-(4-methylpiperazine-1-carbonyl)piperidin-1-yl)phenyl)nicotinamide* (**5n**): yield 45%. ^1^H-NMR (400 MHz, DMSO-*d*_6_) δ 10.25 (s, 1H, NH), 9.13–9.05 (m, 1H, Aromatic hydrogen), 8.78–8.73 (m, 1H, Aromatic hydrogen), 8.32–8.24 (m, 1H, Aromatic hydrogen), 7.56 (dd, *J* = 7.9, 4.8 Hz, 1H, Aromatic hydrogen), 7.41 (s, 1H, Aromatic hydrogen), 7.24–7.14 (m, 2H, Aromatic hydrogen), 6.72 (d, *J* = 8.0 Hz, 1H, Aromatic hydrogen), 3.70 (d, *J* = 12.4 Hz, 2H, piperazine), 3.61–3.49 (m, 4H, piperazine and piperidine), 2.83–2.78 (m, 2H, piperidine), 2.59–2.47 (m, 4H, piperazine), 2.33 (s, 3H, CH_3_), 1.72–1.61 (m, 4H, piperidine). ESI-MS *m*/*z*: 408.1 [M + H]^+^.

*2-Chloro-N-(3-(4-(4-methylpiperazine-1-carbonyl)piperidin-1-yl)phenyl)-4-(methylsulfonyl)benzamide* (**5o**): yield 39%. ^1^H-NMR (400 MHz, CDCl_3_) δ 8.02 (d, *J* = 0.9 Hz, 1H, Ar-H), 7.94 (s, 1H, NH), 7.91–7.86 (m, 2H, Ar-H), 7.41 (s, 1H, Ar-H), 7.23 (d, *J* = 8.1 Hz, 1H, Ar-H), 6.96 (d, *J* = 7.8 Hz, 1H, Ar-H), 6.77 (dd, *J* = 8.2, 1.6 Hz, 1H, Ar-H), 3.78 (d, *J* = 12.5 Hz, 2H, piperazine), 3.66 (s, 2H, piperazine), 3.57 (s, 2H, piperidine), 3.09 (s, 3H, CH_3_), 2.80 (dd, *J* = 12.1, 10.4 Hz, 2H, piperidine), 2.62 (dd, *J* = 7.2, 3.9 Hz, 1H, piperidine), 2.44 (d, *J* = 12.3 Hz, 4H, piperazine), 2.34 (s, 3H, CH_3_), 1.96 (dd, *J* = 12.1, 2.2 Hz, 2H, piperidine), 1.79 (d, *J* = 14.6 Hz, 2H, piperidine). ESI-MS *m*/*z*: 519.1 [M + H]^+^.

*N-(3-(4-(4-methylpiperazine-1-carbonyl)piperidin-1-yl)phenyl)-4-(methylsulfonyl)benzamide* (**5p**): yield 37%. ^1^H-NMR (400 MHz, CDCl_3_) δ 8.06–8.04 (m, 4H, Ar-H), 7.96 (s, 1H, NH), 7.43 (s, 1H, Ar-H), 7.23 (d, *J* = 8.1 Hz, 1H, Ar-H), 6.98 (d, *J* = 7.5 Hz, 1H, Ar-H), 6.77–6.74 (m, 1H, Ar-H), 3.79 (d, *J* = 12.5 Hz, 2H, piperazine), 3.67–3.63 (m, 2H, piperazine), 3.56 (s, 2H, piperidine), 3.09 (s, 3H, CH_3_), 2.84–2.78 (m, 2H, piperidine), 2.65–2.60 (m, 1H, piperidine), 2.44–2.38 (m, 4H, piperazine), 2.33 (s, 3H, CH_3_), 2.00–1.93 (m, 2H, piperidine), 1.83–1.78 (m, 2H, piperidine). ESI-MS *m*/*z*: 485.1 [M + H]^+^.

*N-(3-(4-(4-methylpiperazine-1-carbonyl)piperidin-1-yl)phenyl)-3-(pyridin-2-yl)benzamide* (**5q**): yield 25%. ^1^H-NMR (400 MHz, CDCl_3_) δ 8.72 (d, *J* = 4.5 Hz, 1H, aromatic hydrogen), 8.51 (s, 1H, Aromatic hydrogen), 8.15 (d, *J* = 7.8 Hz, 1H, Aromatic hydrogen), 8.00 (s, 1H, NH), 7.96 (d, *J* = 7.7 Hz, 1H, Aromatic hydrogen), 7.81 (d, *J* = 3.5 Hz, 2H, Aromatic hydrogen), 7.60 (t, *J* = 7.8 Hz, 1H, Aromatic hydrogen), 7.51 (s, 1H, Aromatic hydrogen), 7.31–7.29 (m, 1H, Aromatic hydrogen), 7.23 (d, *J* = 8.1 Hz, 1H, Aromatic hydrogen), 7.01–6.97 (m, 1H, Aromatic hydrogen), 6.76–6.71 (m, 1H, Aromatic hydrogen), 3.80 (d, *J* = 12.3 Hz, 2H, piperazine), 3.65 (d, *J* = 1.2 Hz, 2H, piperazine), 3.55 (d, *J* = 1.1 Hz, 2H, piperidine), 2.79 (dd, *J* = 17.4, 7.1 Hz, 2H, piperidine), 2.65–2.58 (m, 1H, piperidine), 2.41 (dd, *J* = 12.1, 3.6 Hz, 4H, piperazine), 2.32 (s, 3H, CH_3_), 2.05–1.96 (m, 2H, piperidine), 1.82 (d, *J* = 13.9 Hz, 2H, piperidine). ESI-MS *m*/*z*: 484.2 [M + H]^+^.

#### 3.2.3. Procedure for Preparation Intermediates of **6**–**7**

*2-(4-chloro-3-(4-chlorobenzamido)phenyl)acetic acid* (**6**): 2-(3-amino-4-chlorophenyl)acetic acid (1.85 g, 0.01 mol) and triethylamine(3.03 g, 0.03 mol) were dissolved in 10 mL of dichloromethane, then *p*-chlorobenzoyl chloride (1.75 g, 0.01 mol, dissolved in 2 mL dichloromethane) was slowly added to the bottle, and the temperature was controlled at 0 °C. After 2 h of reaction, the reaction was complete. The reaction solution was poured into water and extracted with dichloromethane (30 mL). The reaction mixture was dried, then it was purified by column chromatography on silica gel (dichloromethane:methanol = 20:1) to give product **6** (3.1 g, 96% yield). ^1^H-NMR (400 MHz, DMSO-*d*_6_) δ 12.44 (s, 1H, COOH), 10.17 (s, 1H, NH), 8.11–7.91 (m, 2H, Ar-H), 7.67–7.59 (m, 2H, Ar-H), 7.53–7.43 (m, 2H, Ar-H), 7.21 (dd, *J* = 8.3, 2.2 Hz, 1H, Ar-H), 3.62 (s, 2H, CH_2_).

*tert-butyl-4-(2-(4-chloro-3-(4-chlorobenzamido)phenyl)acetyl)piper-azine-1-carboxylate* (**7**): 1-boc-piperazine (1.02 g, 0.0055 mol), DIPEA (1.93 g, 0.015 mol) were dissolved in 10 mL of tetrahydrofuran, then **6** (1.62 g, 0.005 mol), HOBt (0.68 g, 0.005 mol), EDCI (0.96 g, 0.005 mol) were added to the solution under stirring, the reaction was allowed to react at room temperature for 24 h, the reaction solution was poured into water and extracted with dichloromethane, the reaction solution was washed with saturated sodium bicarbonate solution (10 mL) and 1 mol/L hydrochloric acid solution (10 mL). The reaction solution was dried over anhydrous sodium sulfate and evaporated to dryness under reduced pressure to give a yellow oil which was purified by column chromatography on silica gel (dichloromethane:methanol = 50:1) to get product **7** (2.3 g, 93% yield). ^1^H-NMR (400 MHz, DMSO-*d*_6_) δ 10.14 (s, 1H, NH), 8.00 (d, *J* = 8.6 Hz, 1H, Ar-H), 7.67–7.56 (m, 2H, Ar-H), 7.51–7.42 (m, 2H, Ar-H), 7.17 (dd, *J* = 8.3, 2.2 Hz, 1H, Ar-H), 3.77 (s, 2H, CH_2_), 3.52–3.40 (m, 4H, piperazine), 3.29 (d, *J* = 5.4 Hz, 4H, piperazine), 1.40 (s, 9H, CH_3_). 

#### 3.2.4. General Procedure for Preparation of Target Compounds of **8a**–**c**

To a solution of **7** (0.12 g, 0.00025 mol) dissolved in CH_2_Cl_2_ (2 mL) was added trifluoroacetic acid (TFA, 3 mL) and the solution stirred at room temperature for 24 h. The solution was poured into water, it was adjusted to pH = 11, the white product was obtained by suction filtration, it did not need to be further purified. The product and triethylamine (0.076 g, 0.00075 mol) were dissolved in 5 mL of dichloromethane, then sulfonyl chloride (0.00025 mol, dissolved in 2 mL dichloromethane) was slowly added to the bottle, and the temperature was controlled at 0 °C. After 2 h of reaction, the reaction was complete. The reaction solution was poured into water and extracted with dichloromethane (20 mL). The reaction mixture was dried, and then it was purified by column chromatography on silica gel to give product **8a**–**c**.

Compounds **8a**–**c** was synthesized according to the above steps. The spectral data are as follows:

*4-Chloro-N-(2-chloro-5-(2-(4-(methylsulfonyl)piperazin-1-yl)-2-oxoethyl)phenyl)benzamide* (**8a**): yield 85.7%. ^1^H-NMR (400 MHz, DMSO-*d*_6_) δ10.16 (s, 1H, NH), 8.00 (d, *J* = 8.5 Hz, 2H, Ar-H), 7.62 (d, *J* = 8.5 Hz, 2H, Ar-H), 7.55−7.39 (m, 2H, Ar-H), 7.17 (d, *J* = 8.2 Hz, 1H, Ar-H), 3.80 (s, 2H, CH_2_), 3.60 (d, *J* = 29.6 Hz, 4H, piperazine), 3.14–3.04 (m, 4H, piperazine), 2.88 (s, 3H, CH_3_). ESI-MS *m*/*z*: 470.0 [M + H]^+^.

*4-Chloro-N-(2-chloro-5-(2-(4-((4-fluorophenyl)sulfonyl)piperazin-1-yl)-2-oxoethyl)phenyl)benzamide* (**8b**): yield 88.4%. ^1^H-NMR (400 MHz, DMSO-*d*_6_) δ 10.10 (s, 1H, NH), 7.98 (d, *J* = 8.6 Hz, 2H, Ar-H), 7.79 (dd, *J* = 8.8, 5.1 Hz, 2H, Ar-H), 7.62 (d, *J* = 8.6 Hz, 2H, Ar-H), 7.47 (t, *J* = 8.8 Hz, 2H, Ar-H), 7.42 (d, *J* = 8.3 Hz, 1H, Ar-H), 7.39 (s, 1H, Ar-H), 7.07 (d, *J* = 10.2 Hz, 1H, Ar-H), 3.71 (s, 2H, CH_2_), 3.58 (d, *J* = 23.8 Hz, 4H, piperazine), 2.86 (d, *J* = 19.6 Hz, 4H, piperazine). ESI-MS *m*/*z*: 550.0 [M + H]^+^.

*N-(5-(2-(4-(butylsulfonyl)piperazin-1-yl)-2-oxoethyl)-2-chlorophenyl)-4-chlorobenzamide* (**8c**): yield 90.1%. ^1^H-NMR (400 MHz, DMSO-*d*_6_) δ 10.16 (s, 1H, NH), 8.00 (d, *J* = 8.4 Hz, 2H, Ar-H), 7.62 (d, *J* = 8.4 Hz, 2H, Ar-H), 7.50 (d, *J* = 8.2 Hz, 1H, Ar-H), 7.46 (s, 1H, Ar-H), 7.17 (d, *J* = 9.4 Hz, 1H, Ar-H), 3.79 (s, 2H, CH_2_), 3.58 (d, *J* = 22.1 Hz, 4H, piperazine), 3.14 (dd, *J* = 15.1, 4.5 Hz, 4H, piperazine), 3.06–2.98 (m, 2H, CH_2_), 1.62 (p, *J* = 7.7 Hz, 2H, CH_2_), 1.36 (h, *J* = 7.3 Hz, 2H, CH_2_), 0.87 (t, *J* = 7.3 Hz, 3H, CH_3_). ESI-MS *m*/*z*: 512.0 [M + H]^+^.

### 3.3. Biological Methods

#### 3.3.1. NIH3T3-Gli-Luciferase Reporter Assay

##### Cell Culture

NIH3T3 cells (Chinese Academy of Sciences cell bank, Mouse embryonic fibroblast cell line) were grown in Dulbecco’s Modified Eagle Media (DMEM) media supplemented with 10% fetal bovine serum (FBS) at 37 °C with a humidified 5% CO_2_ atmosphere.

##### Plating Cells

NIH3T3 cells were plated one day before transfection. To prepare NIH3T3 cells, enough cells were collected to complete the transfection experiment, and centrifuged for 5 min at 300× *g*. The cell pellet was resuspended to an appropriate concentration in DMEM media with 0.5% FBS, then 5000 cells were plated in 100 μL per well of a 96-well plate.

##### Transfection

The NIH3T3 cells were transfected with the Glioma-associated oncogene (GLI)-reporter, negative control, and positive control constructs (QIAGEN, Cat# CCS-6030L) by using Fugene HD transfection reagents (Promega, Cat. No. E2311, Madison, WI). The FuGENER HD Transfection Reagent/DNA mixture was incubated for 15 min at room temperature. A total of 10 μL of the FuGENER HD Transfection Reagent/DNA mixture was added per well to the 96-well plate containing 100 μL cells in growth medium. The mixture was mixed gently using a plate shaker. Cells were returned to the incubator for 24–48 h. Transfection efficiency was estimated by observing green fluorescent protein (GFP) expression in the positive control wells by fluorescence microscopy.

##### Compound Treatment

After 48 h of transfection, culture medium was to fresh assay medium (Opti-MEM^®^ containing 0.5% of FBS), and cells were treated with 10 nM of smoothened agonist (SAG) (MedChem Express, Cat# HY-12848, as the agonist of Hh signaling) and 1 μM of test compounds at a final concentration. Cells were incubated with SAG and compounds for 24 h. To study the effect of test compounds, cells were harvested 24 h after treatment to perform the dual-luciferase assay.

##### Dual-Luciferase Assay

Dual-Luciferase assays were conducted using the Dual-Luciferase^®^ Assay System (Promega, Cat# E1910) according to Promega’s protocol. Each system included: 5X Passive Lysis Buffer (PLB), Luciferase Assay Buffer II, Luciferase Assay Substrate, and 50X Stop & Glo^®^ Substrate and Stop & Glo^®^ Buffer.

After 24 h treatment, the assay medium was removed and cells were washed with PBS buffer, and then 20 μL of 1XPLB was added to each well. The culture plates were incubated on a plate shaker with gentle shaking at room temperature for 15 min. A total of 100 μL LARII was added to each well, and the firefly luciferase luminescence signal was measured using an EnVision^®^ 2104 Multilabel Reader (PerkinElmer, Waltham, MA, USA), with a measure time of 10s. A total of 100 μL Stop & Glo^®^ Reagent was added to each well, and Renilla luciferase luminescence signal was measured using an EnVision^®^ 2104 Multilabel Reader (PerkinElmer); the measure time was 10 s.

##### Data Analysis

Relative Luciferase Units (RLU) Calculation

Firefly: Renilla activity ratios were generated from experimental and control transfections. Ratios from transcription factor-responsive reporter transfections were divided by ratios from negative control transfections to obtain relative luciferase units.

The inhibition rate of the compounds was calculated as follows:

% inhibition = [(RLU of control well−RLU of test compound well)/RLU of control well] × 100%

The histogram of % inhibition of compounds was generated by using GraphPad Prism 5.0 software (GraphPad Software Inc., La Jolla, CA, USA).

#### 3.3.2. Hsmo-BC Binding Assay

BODIPY-cyclopamine binding assay is a fluorescence-based assay used to analyze the binding of Smo agonists/antagonists. HEK293 cells were transfected with pCMV-HA/hSmo using Lipofectamine reagent (Lipofectamine™ 2000, Invitrogen, Carlsbad, CA, USA). Briefly, 50 μL of Lipofectamine 2000 and 22.5 μg of pCMV-HA/hSmo were each incubated in 1.5 mL of Opti-MEMI media (Invitrogen) at room temperature for 30 min and then was added to 1 × 10^6^ HeK293 cells in 25 mL of culture medium in a 75 cm^2^ cell culture flask. After overnight incubation under 5% CO_2_ at 37 °C, cells were detached using Versene (Invitrogen) and resuspended in culture medium. The suspended cells were added to a 96-well plate at 10,000 cells/well. After removing the media from each well, the cells were fixed with 4% paraformaldehyde (PFA) for 20 min at room temperature. Then the PFA buffer was removed, and the cells were incubated with 4′,6-diamidino-2-phenylindole (DAPI) (5 mg/mL) for 10 min, followed by washing with PBS four times. Subsequently, cells were incubated for 3 h at room temperature in PBS containing 100 nM BODIPY-cyclopamine and serial diluted compounds (1000–0.02 nM in DMSO/H_2_O) for competitive binding. After incubation, the cells were washed five times with PBST (PBS buffer supplied with 0.05% Tween-20). The fluorescence images were automatically captured and analyzed by a high content fluorescence imaging system (Arrayscan VTI, Thermo, Waltham, MA, USA). Data was expressed as a percentage of fluorescence intensity observed with BODIPY-cyclopamine alone. Vismodegib was used as a reference compound to normalize the data. IC_50_ values were calculated with GraphPad Prism software using the sigmoidal dose-response function.

## 4. Conclusions

Based on the structure of the compound MRT-10, two series of target compounds were designed by analyzing the structure of the Hh signaling pathway, which was listed or already in clinical trial. The Gh-luc double luciferase gene model was used as the experimental evaluation system and the Hh signal pathway inhibition activity was tested on the 20 synthesized compounds with Vismodegib as the positive control drug. The results showed that compounds **5l** and **5q** are preferred compounds. The activities of the compounds were favorable when the phenyl substituent was an electron-donating substituent. Smo binding experiments were carried out on compounds with better activity in luciferase experiments. The results showed that the inhibitory activity of these compounds on Smo receptor was positively correlated with the activity of Hh signaling pathway, indicating that the target compound inhibited the activity of Hh signaling pathway by binding to Smo receptor.

## Figures and Tables

**Table 1:**
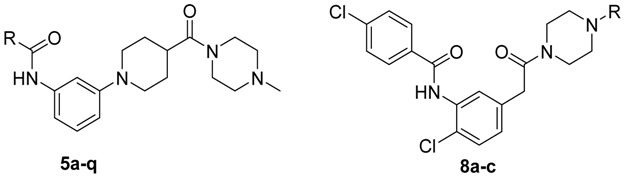
The structures and the hedgehog pathway inhibition of target compounds.

Compd.	R	Gli-luc Reporter IC_50_^ a ^(μmol/L)	Compd.	R	Gli-luc Reporter IC_50_^ a ^(μmol/L)
**5a**	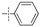	28.52 ± 0.02	**5l**	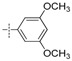	1.83 ± 0.03
**5b**	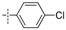	12.47 ± 0.11	**5m**	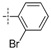	10.49 ± 0.05
**5c**	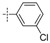	14.63 ± 0.01	**5n**	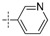	5.32 ± 0.13
**5d**	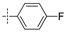	15.51 ± 0.03	**5o**	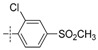	4.28 ± 0.01
**5e**	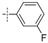	17.59 ± 0.15	**5p**	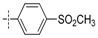	3.43 ± 0.08
**5f**	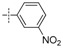	53.34 ± 0.07	**5q**	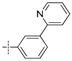	1.26 ± 0.03
**5g**	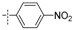	60.56 ± 0.04	**8a**	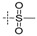	6.88 ± 0.13
**5h**	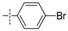	7.85 ± 0.01	**8b**	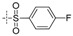	12.7 ± 0.16
**5i**	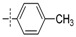	16.32 ± 0.05	**8c**	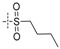	8.62 ± 0.09
**5j**	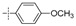	4.74 ± 0.02	MRT10 ^b^		2.15
**5k**	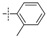	16.61 ± 0.12	Vismodegib ^b^		0.025

^a^ The values are an average of triplicate separate determinations. ^b^ Used as a positive control.

**Table 2:** In vitro inhibition of Smo for selected derivatives.

Compound	Smo-BCB IC_50_^a^ (μM)
**5l**	2.41 ± 0.01
**5n**	6.35 ± 0.07
**5o**	6.23 ± 0.12
**5q**	1.61 ± 0.02
**Vismodegib** ^b^	0.0051

^a^ Inhibition of BODIPY-cyclopamine fluorescence signaling in the competitive displacement experiment using HEK 293 cells over-expressing human Smo. Data are expressed from a single IC_50_ determination. ^b^ Used as a positive control.

## Supplementary Materials

Supplementary materials are available online. MS and ^1^H-NMR spectra of the intermediates of **1**–**4**, **6**–**7** and compounds **5a**–**q**, **8a**–**c**.
