# Supplementary material for: Synthesis and Smo Activity of Some Novel Benzamide Derivatives
Source: Molecules. 2017 Dec 31;23(1):85. doi: 10.3390/molecules23010085 (PMC6017536; doi:10.3390/molecules23010085)
Supplement: Supplementary file 1 [file molecules-23-00085-s001.pdf]

Supporting Information

Synthesis and Antiproliferative Activity of Novel Benzamide Derivatives

Huaiwei Ding [1], Kai Chen[1], Bingke Song[1], Chenglong Deng[1], Wei Li [1], Li Niu [1], Mengxuan Bai [1], Hongrui Song [1,*] and Lijuan Zhang[1,*]

[1] Key Laboratory of Structure-Based Drug Design and Discovery, Ministry of Education, Shenyang Pharmaceutical University, Shenyang 110016, China; dinghuaiwei627@163.com (H.D.); kylechen0322@163.com (K.C.); s13050245586@163.com (B.S.); m15694145159@163.com (C.D.); 15757196069@163.com (W.L.); 18435154073@163.com (L.N.); baimengxuan_2015@126.com (M.B.)

* Correspondence: hongruisong0314@163.com (H.S.), yd_zlj@163.com (L.Z.)

Spectrum of intermediates and the target compounds

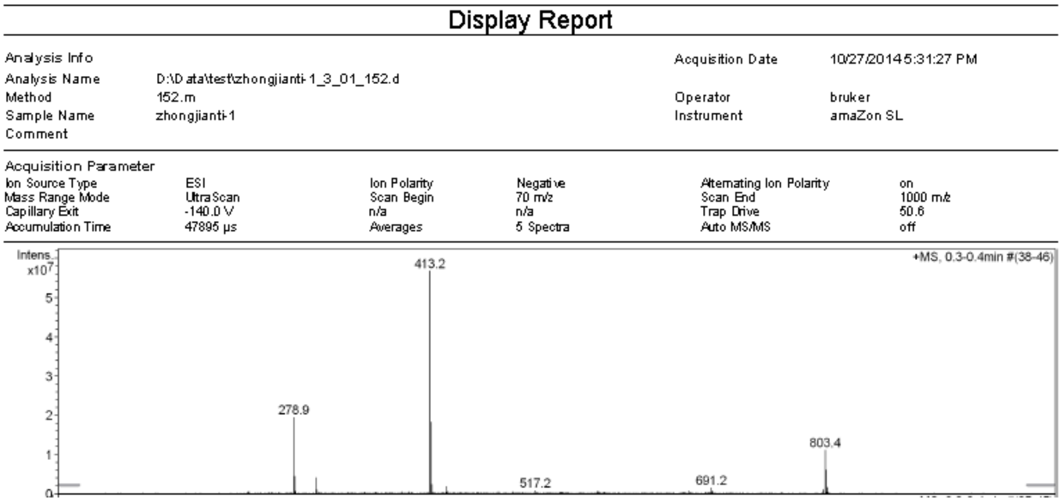

Fig. 1-1 MS spectrum of compound **1**

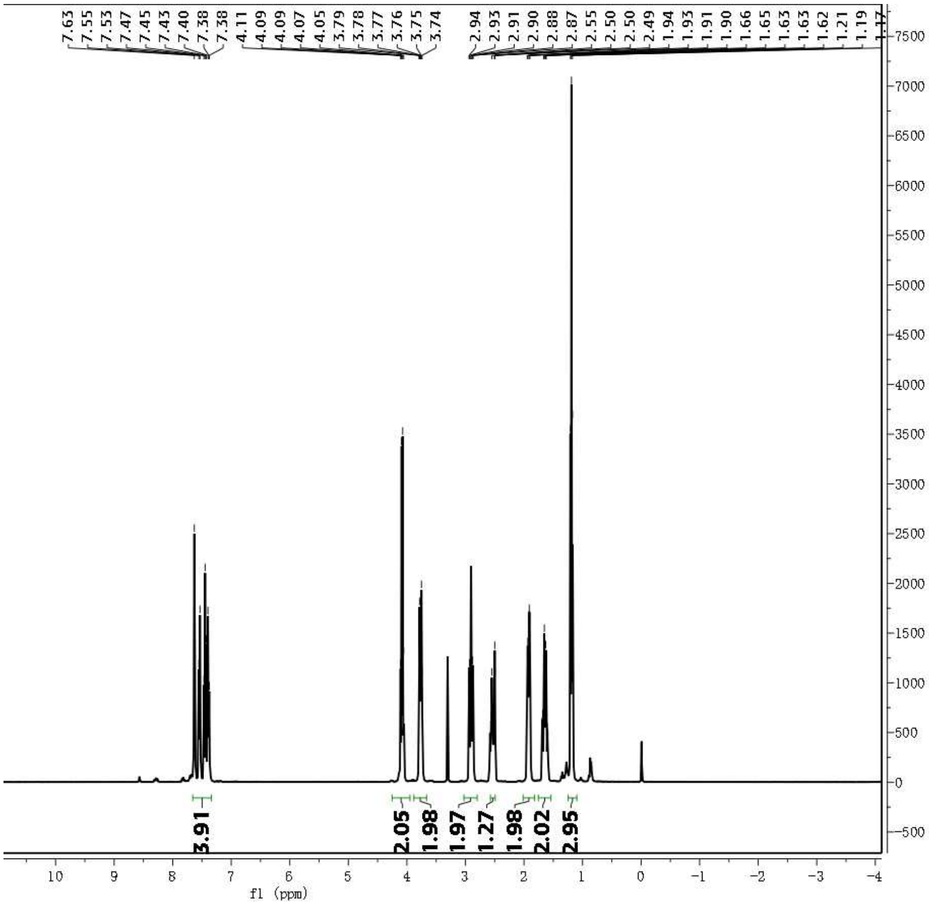

Fig. 1-2 ^{1}H-NMR spectrum of compound **1**

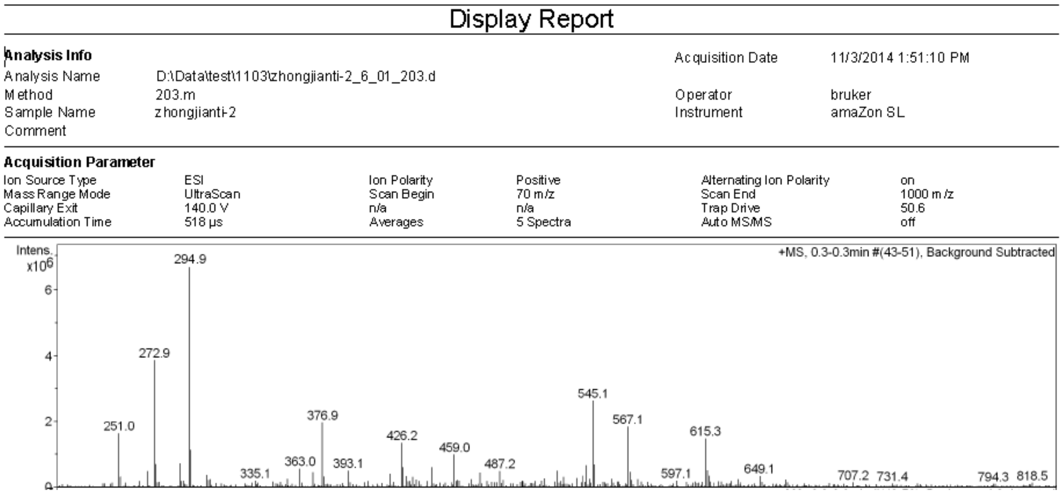

Fig. 2-1 MS spectrum of compound **2**

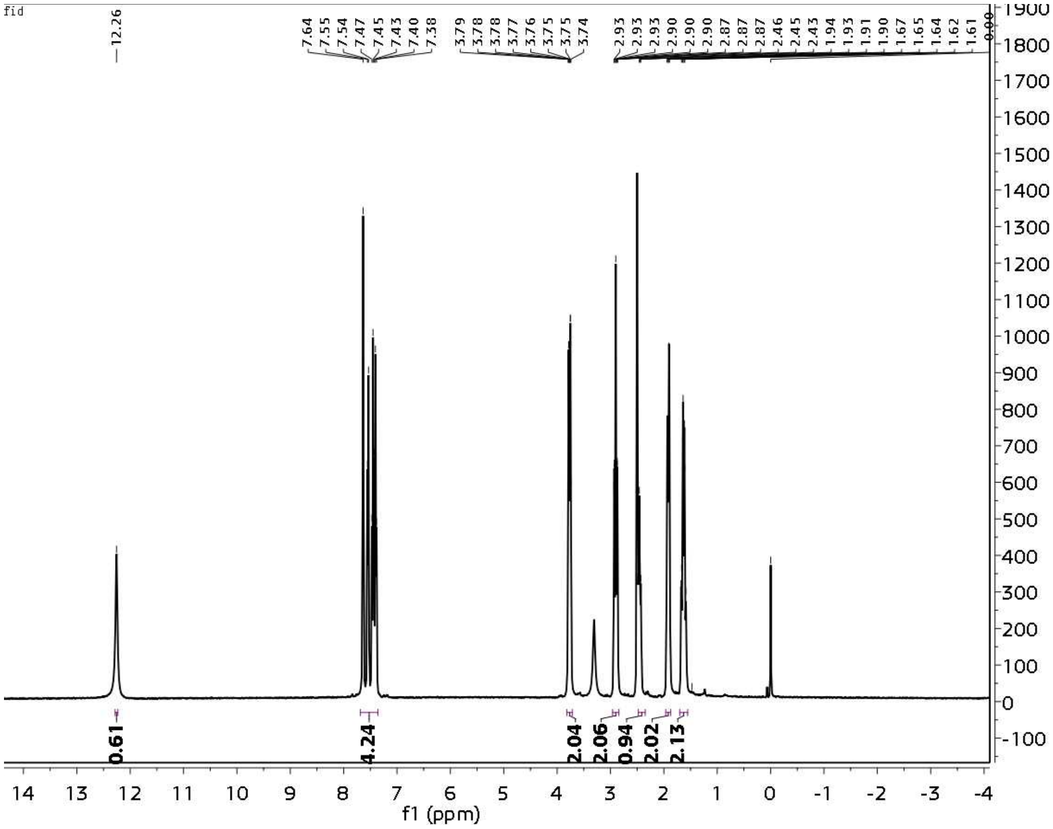

Fig. 2-2 ^{1}H-NMR spectrum of compound **2**

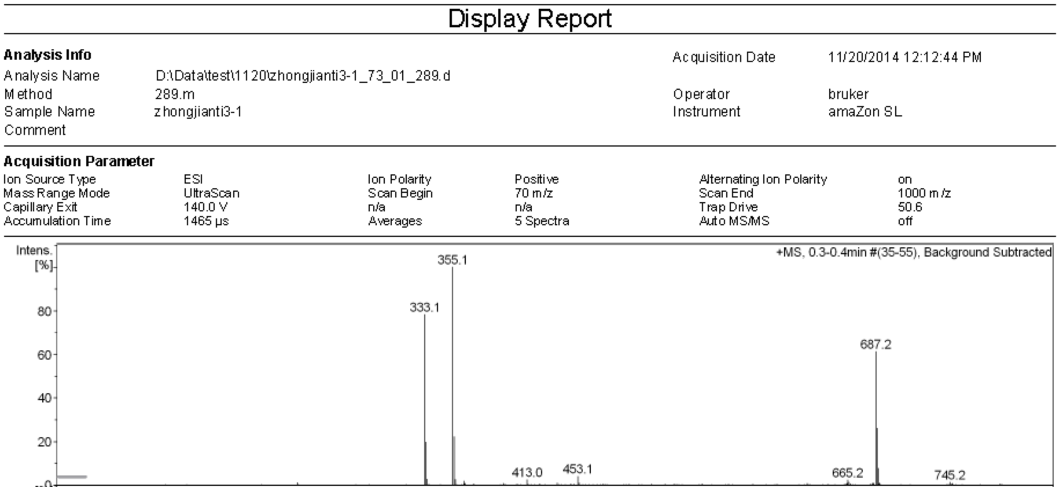

Fig. 3-1 MS spectrum of compound **3**

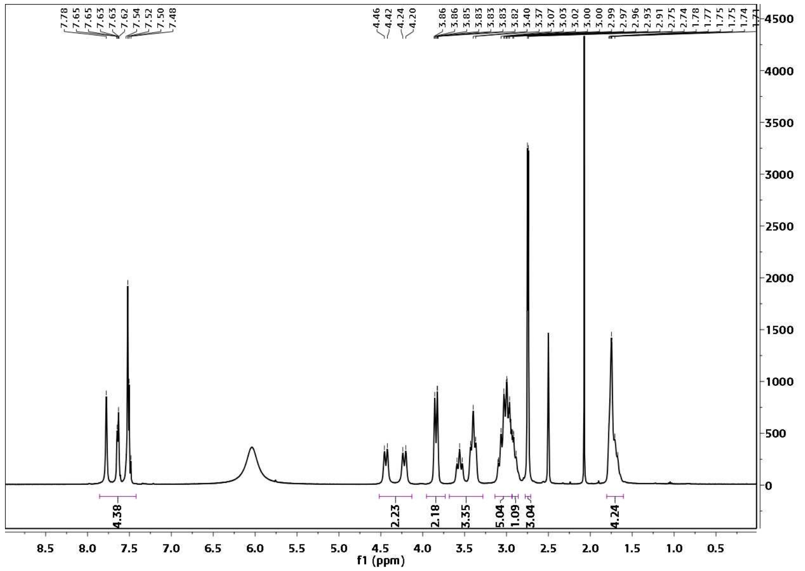

Fig. 3-2 ^{1}H-NMR spectrum of compound **3**

Direct Mass Spectrometry Analysis

Analysis Name: 17011178.d **Instrument:** LC-MSD-Trap-SL **Print Date:** 1/11/2017 7:18:21 PM
Sample Name: CK-1 **Operator:** Administrator **Acq. Date:** 1/11/2017 7:16:52 PM

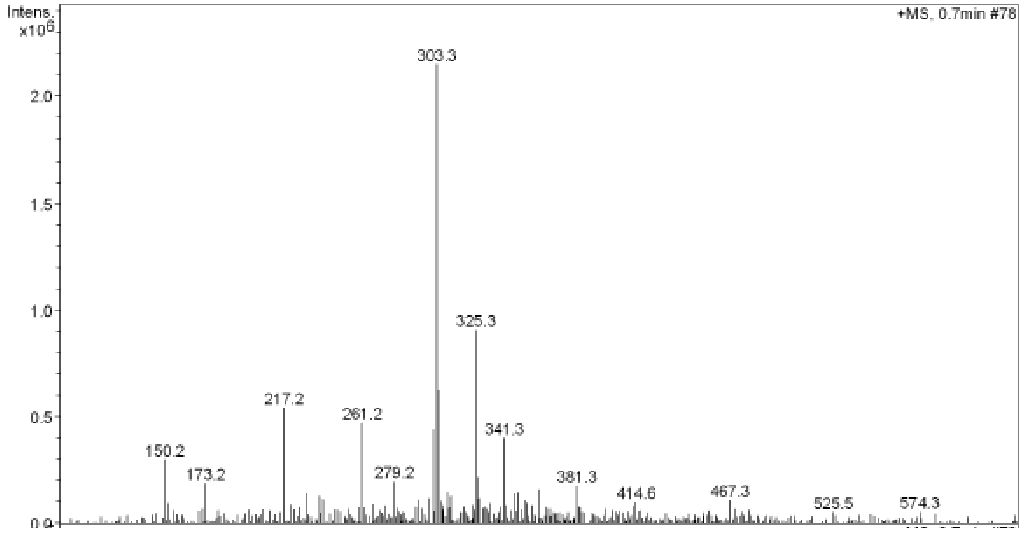

Fig. 4-1 MS spectrum of compound **4**

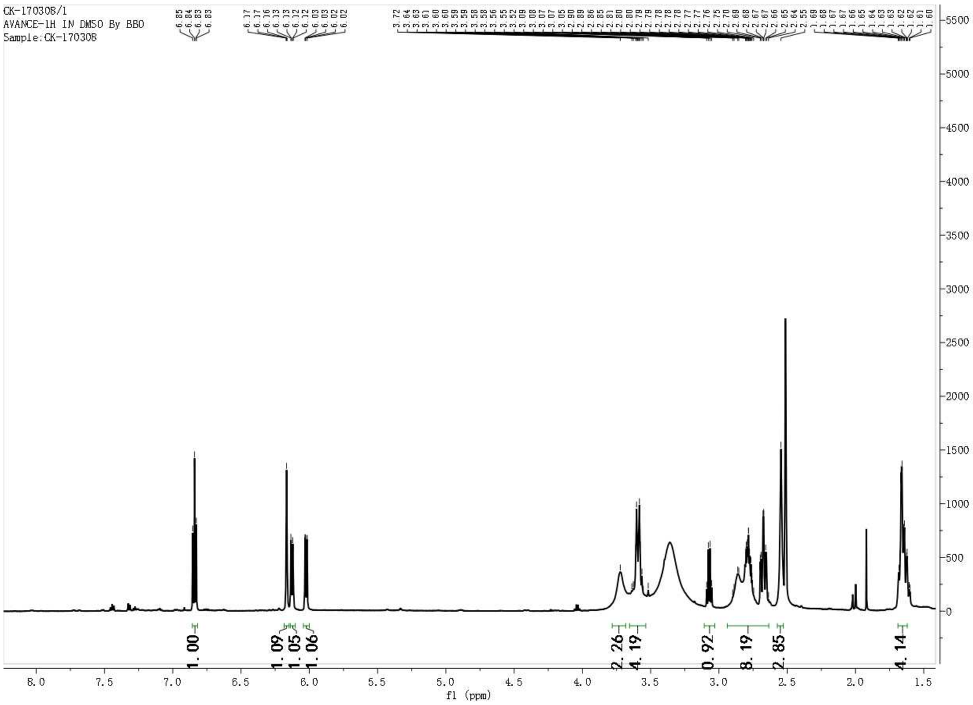

Fig. 4-2 ^{1}H-NMR spectrum of compound **4**

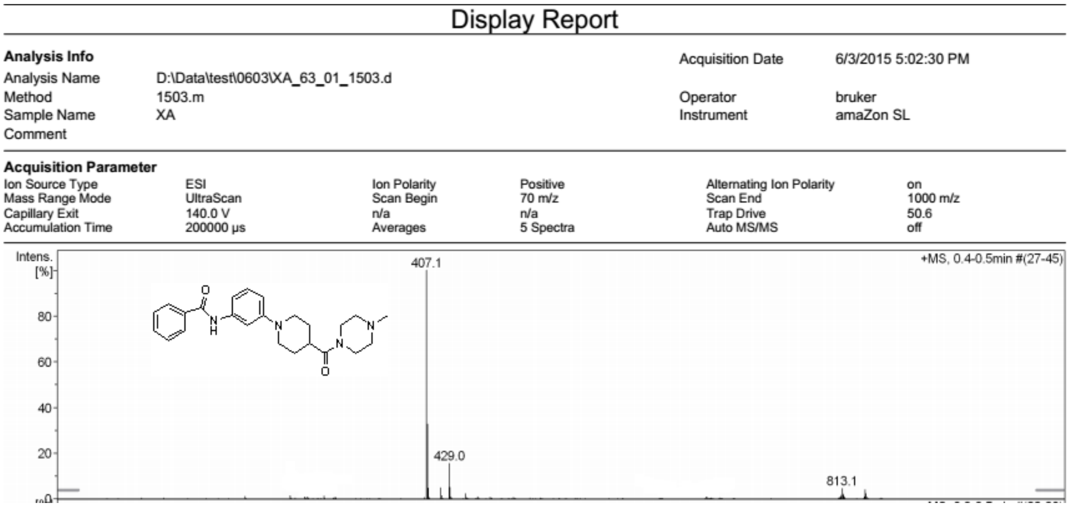

Fig. 5-1 MS spectrum of compound **5a**

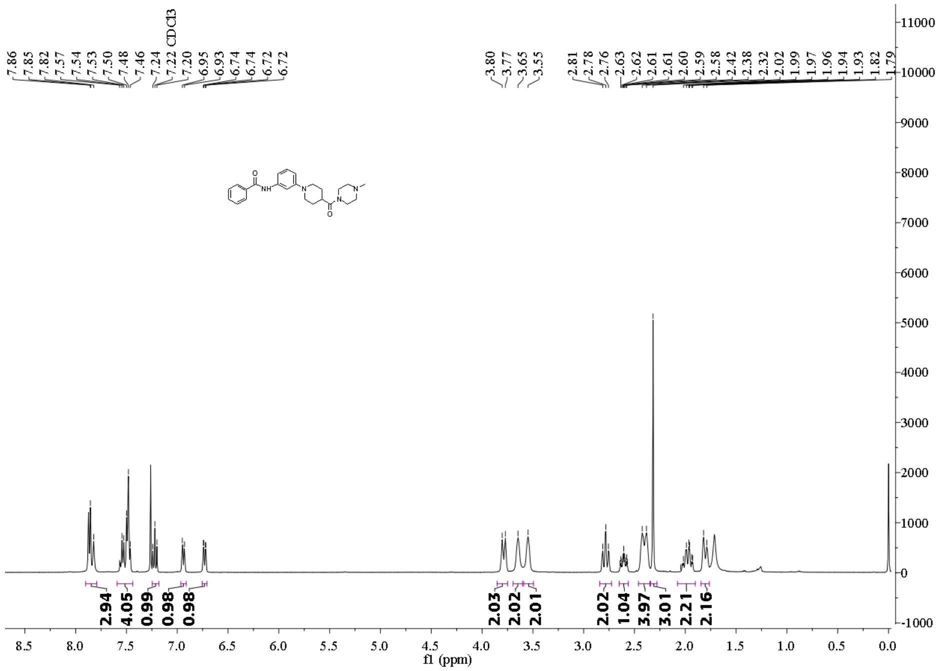

Fig. 5-2 ^{1}H-NMR spectrum of compound **5a**

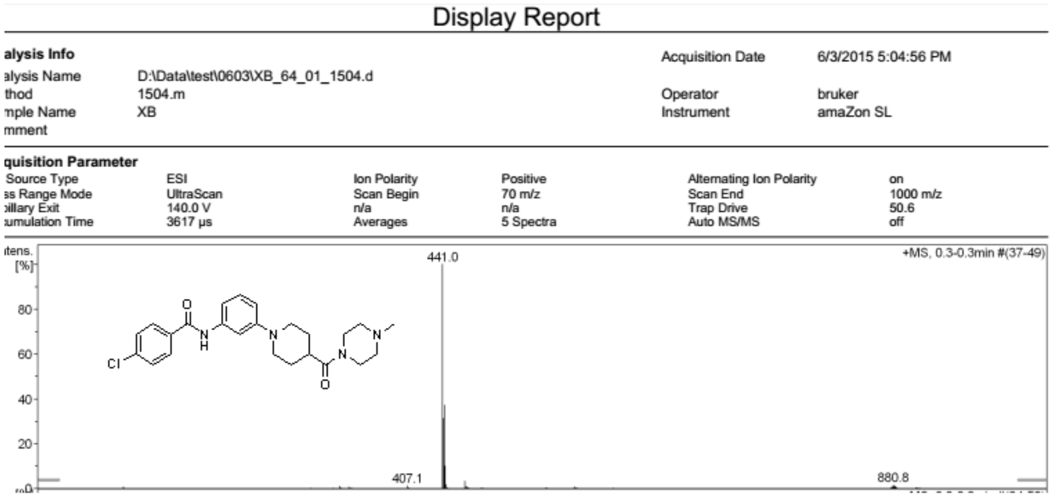

Fig.6-1 MS spectrum of compound **5b**

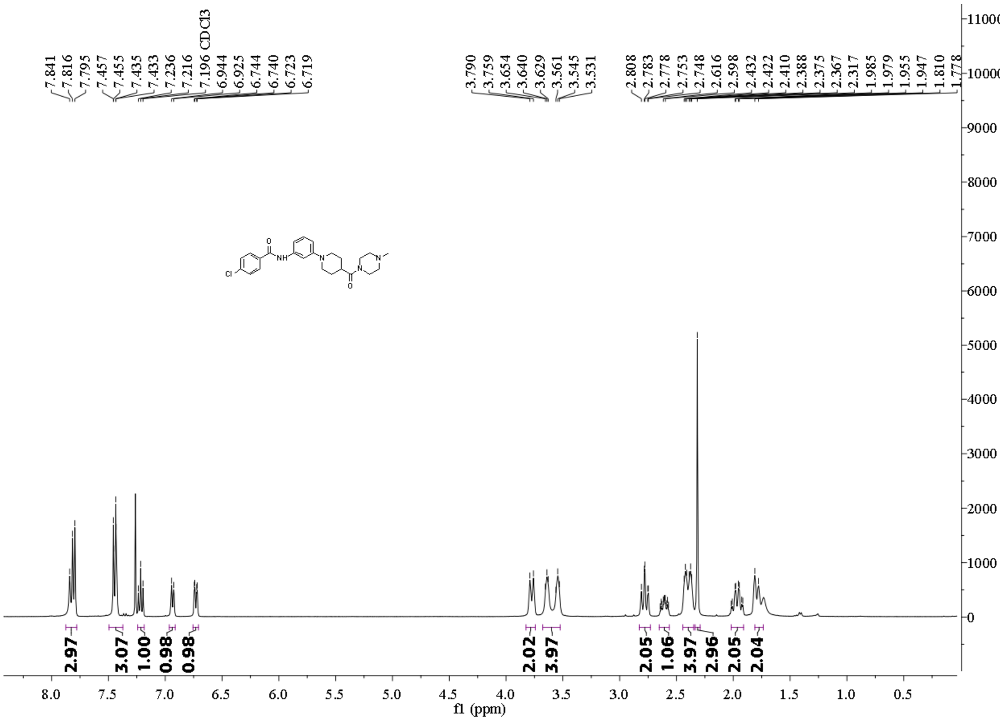

Fig. 6-2 ^{1}H-NMR spectrum of compound **5b**

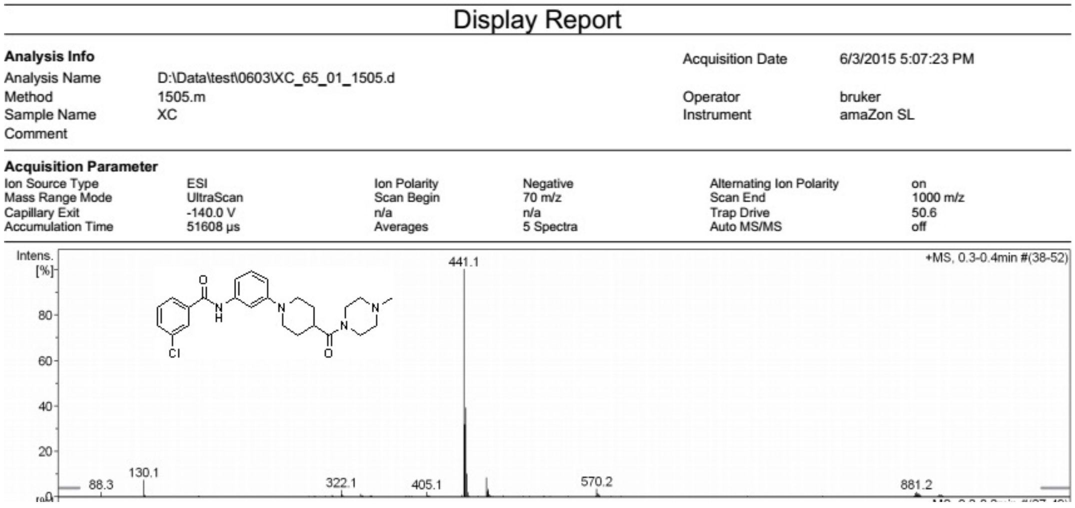

Fig.7-1 MS spectrum of compound **5c**

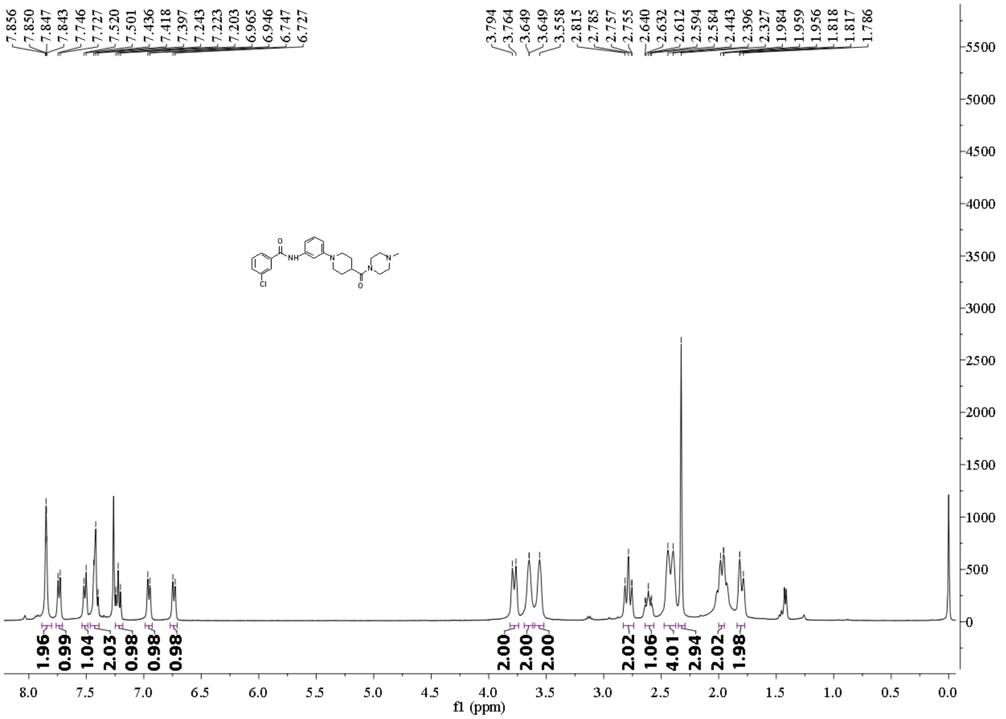

Fig. 7-2 ^{1}H-NMR spectrum of compound **5c**

Display Report

Analysis Info

Analysis Name	D:\Data\test\0603\XD_66_01_1506.d	Acquisition Date	6/3/2015 5:09:49 PM
Method	1506.m	Operator	bruker
Sample Name	XD	Instrument	amaZon SL
Comment			

Acquisition Parameter

Ion Source Type	ESI	Ion Polarity	Positive	Alternating Ion Polarity	on
Mass Range Mode	UltraScan	Scan Begin	70 m/z	Scan End	1000 m/z
Capillary Exit	140.0 V	n/a	n/a	Trap Drive	50.6
Accumulation Time	3739 µs	Averages	5 Spectra	Auto MS/MS	off

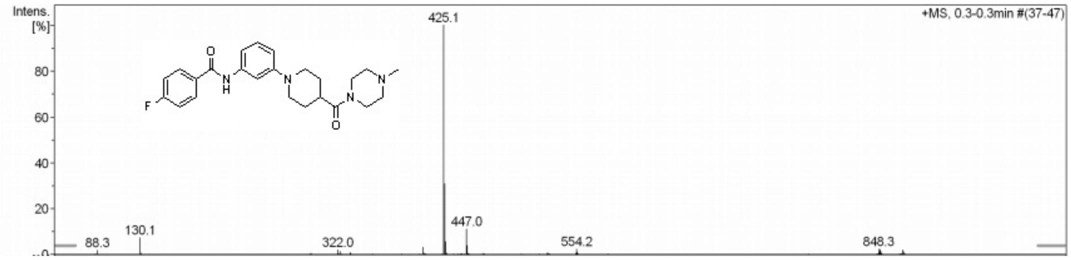

Fig.8-1 MS spectrum of compound **5d**

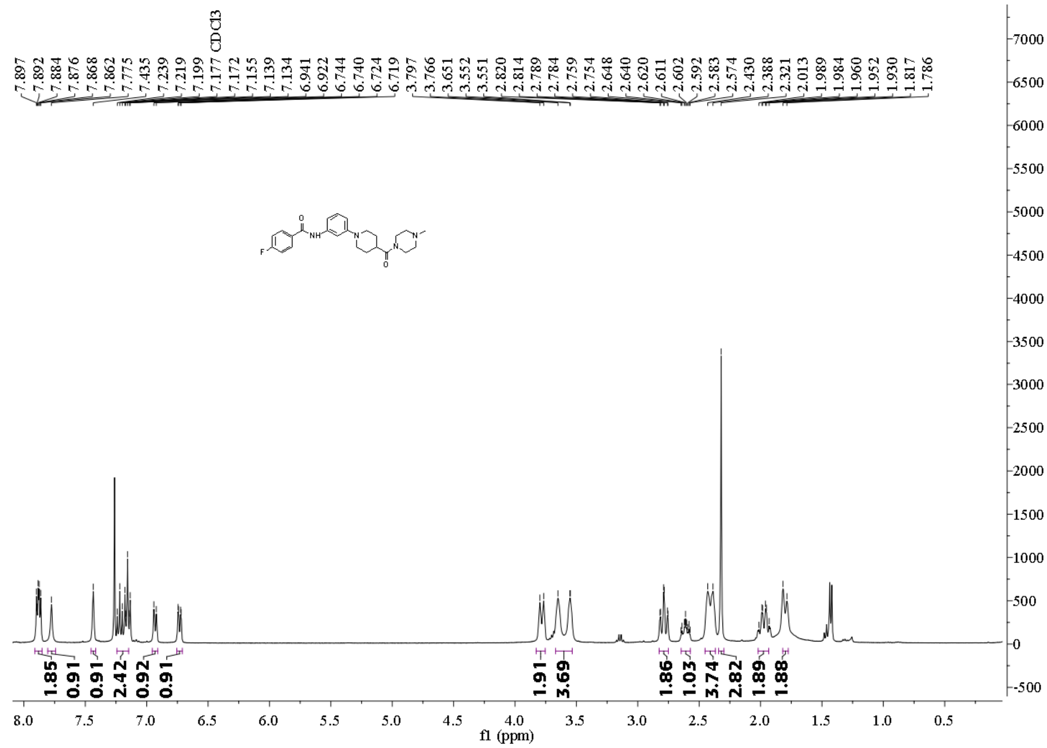

Fig.8-2 ^{1}H-NMR spectrum of compound **5d**

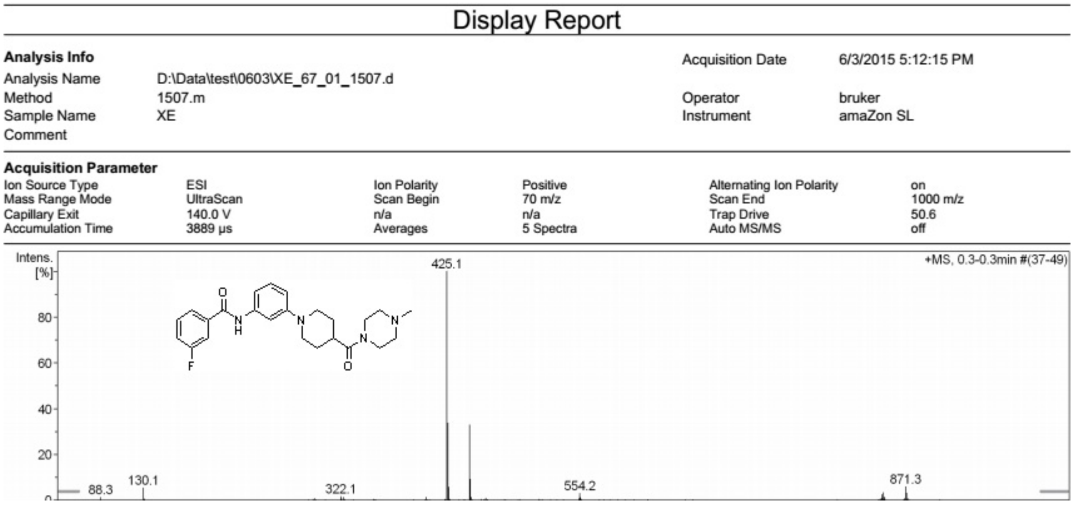

Fig.9-1 MS spectrum of compound **5e**

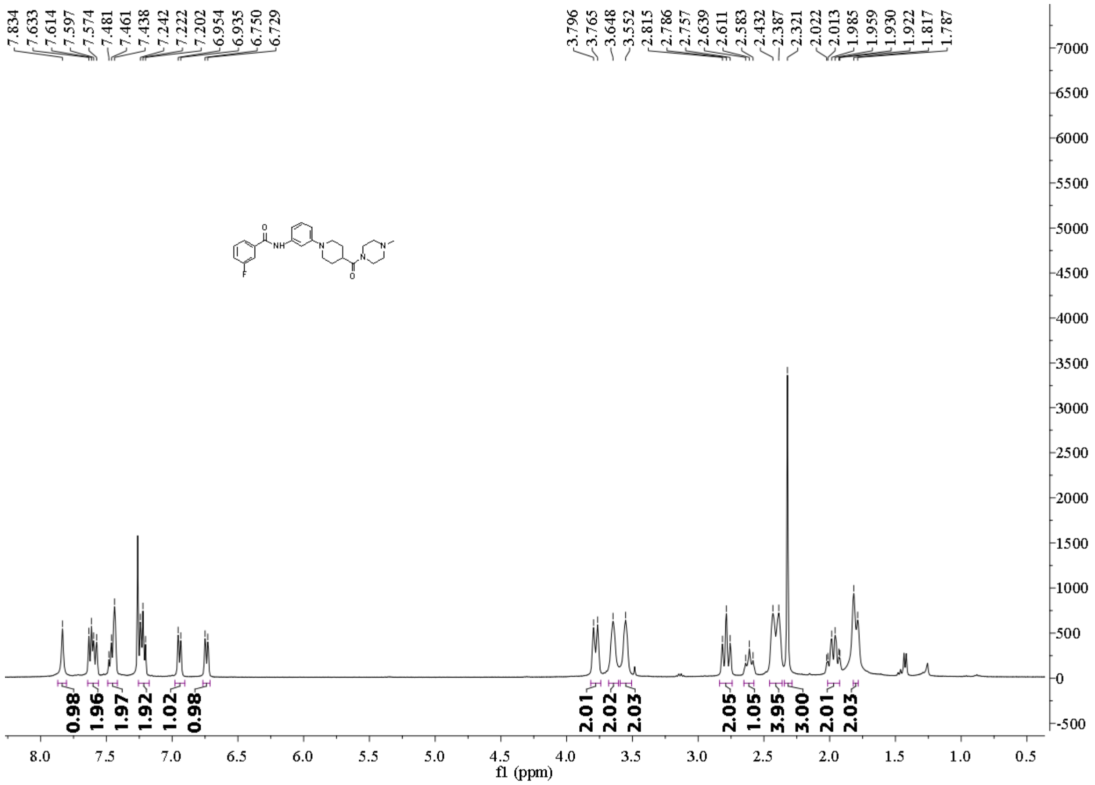

Fig.9-2 ^{1}H-NMR spectrum of compound **5e**

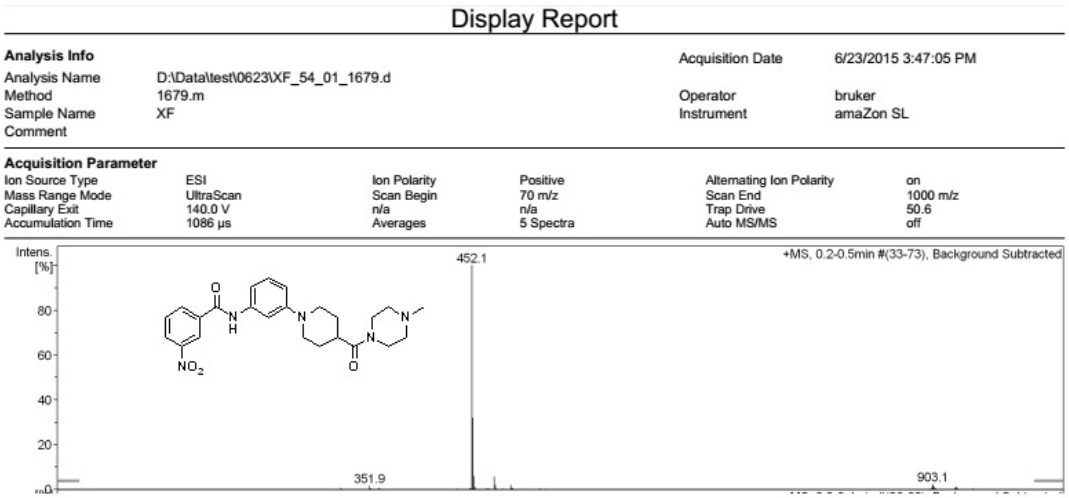

Fig.10-1 MS spectrum of compound **5f**

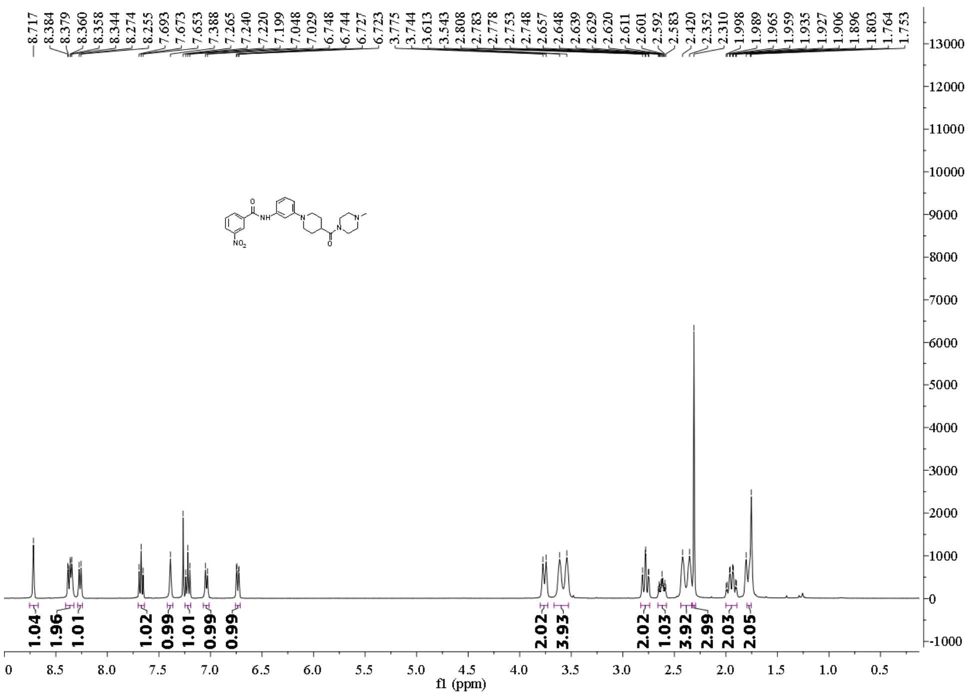

Fig.10-2 ^{1}H-NMR spectrum of compound **5f**

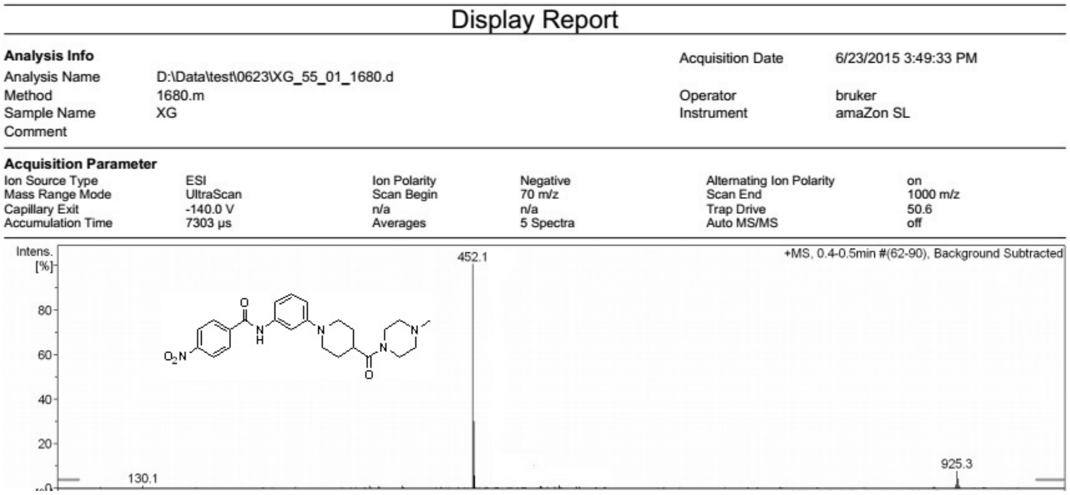

Fig.11-1 MS spectrum of compound **5g**

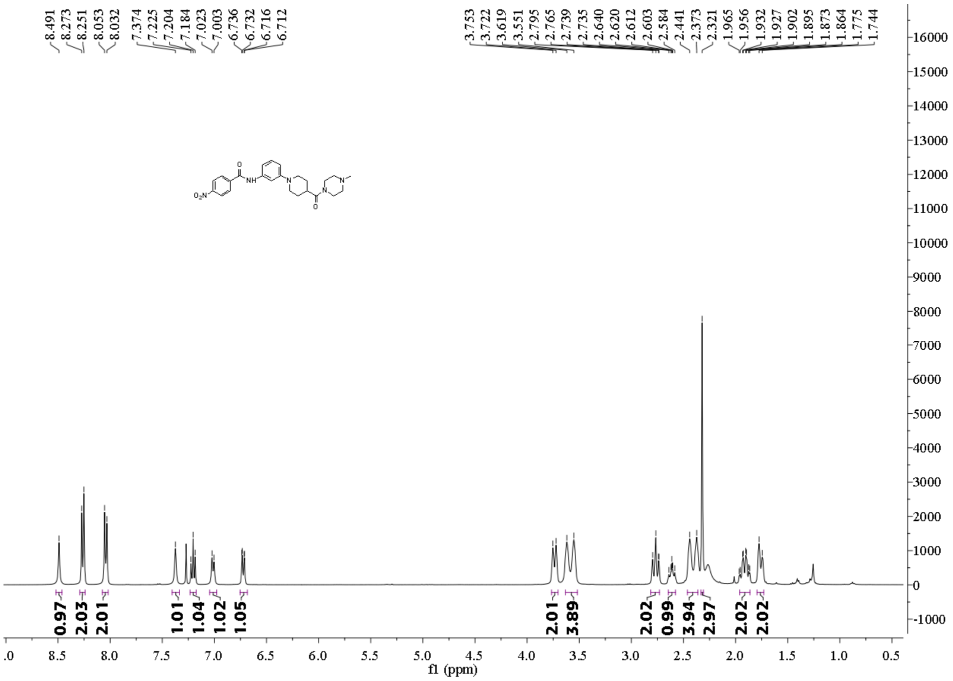

Fig.11-2 ^{1}H-NMR spectrum of compound **5g**

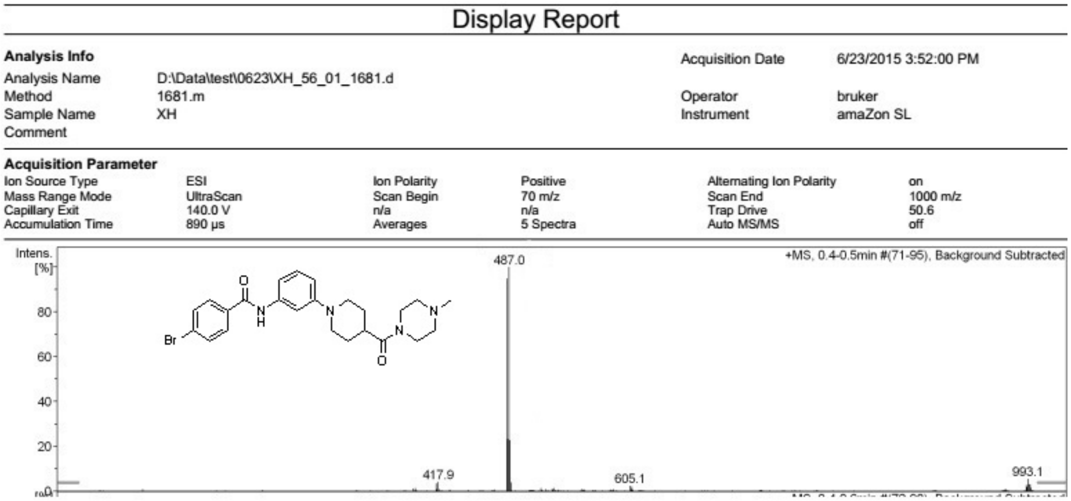

Fig.12-1 MS spectrum of compound **5h**

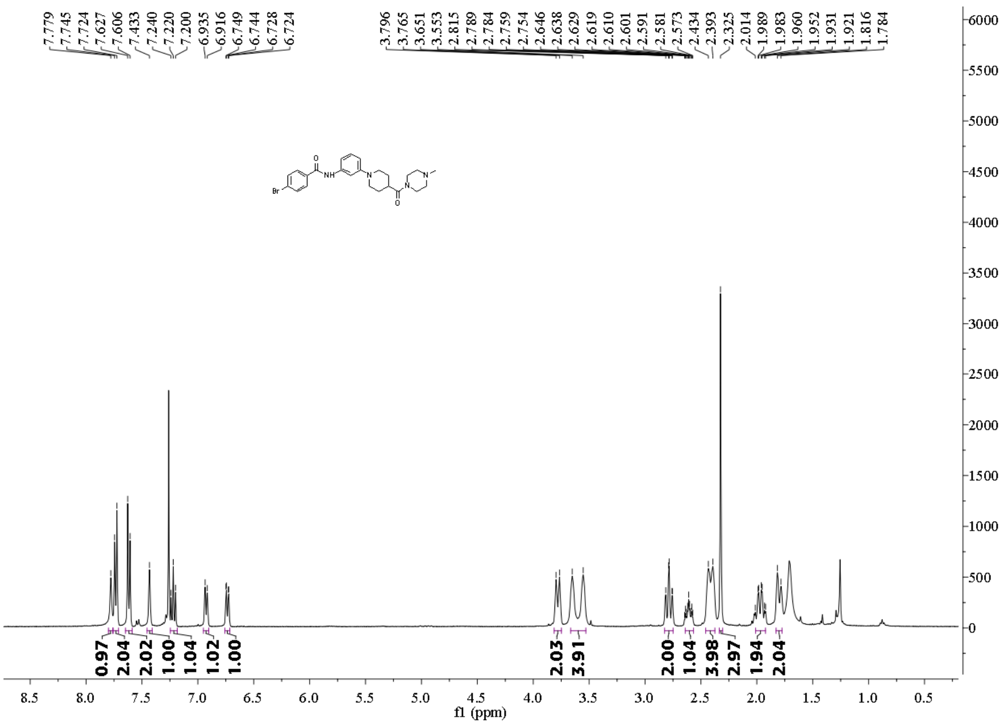

Fig.12-2 ^{1}H-NMR spectrum of compound **5h**

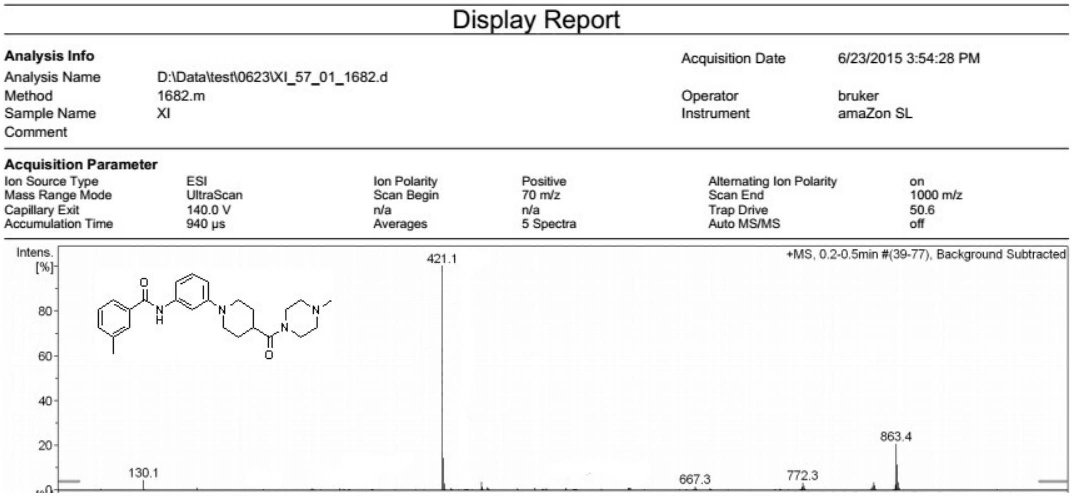

Fig.13-1 MS spectrum of compound **5i**

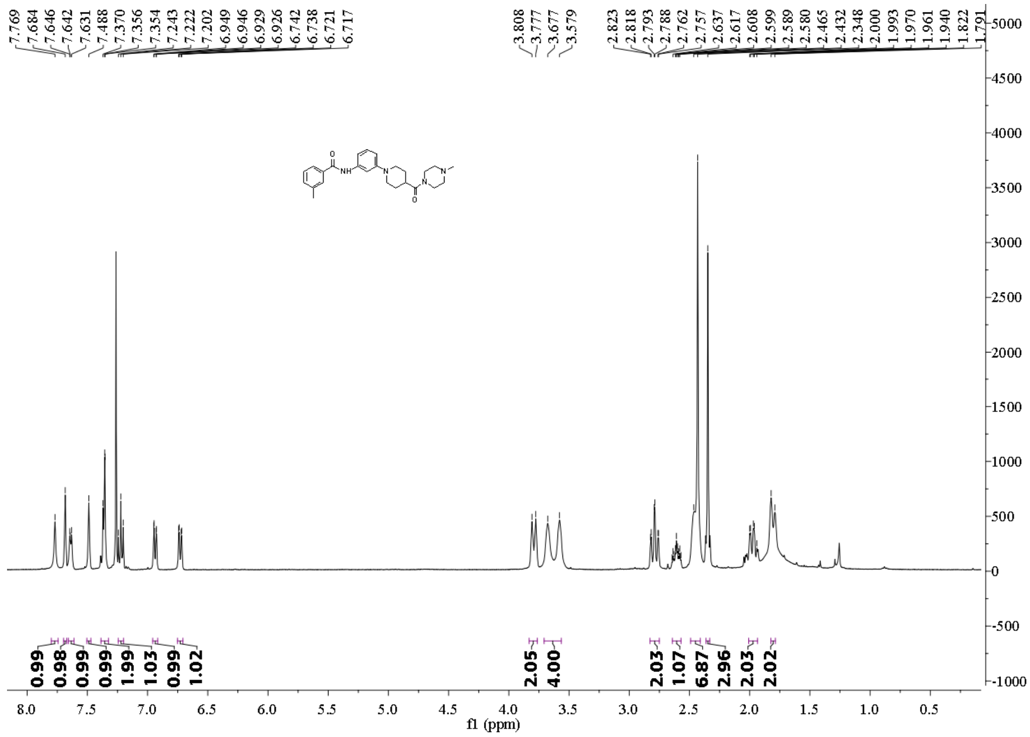

Fig.13-2 ^{1}H-NMR spectrum of compound **5i**

Display Report

Analysis Info

Analysis Name	D:\Data\test\0623\XJ_58_01_1683.d	Acquisition Date	6/23/2015 3:56:57 PM
Method	1683.m	Operator	bruker
Sample Name	XJ	Instrument	amaZon SL
Comment			

Acquisition Parameter

Ion Source Type	ESI	Ion Polarity	Positive	Alternating Ion Polarity	on		
Mass Range Mode	UltraScan	Scan Begin	70 m/z	Scan End	1000 m/z		
Capillary Exit	140.0 V	n/a	n/a	Trap Drive	50.6		
Accumulation Time	764 µs	Averages	5 Spectra	Auto MS/MS	off		

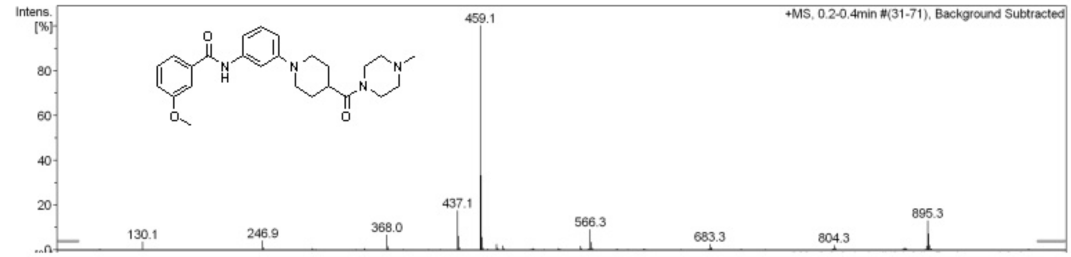

Fig.14-1 MS spectrum of compound **5j**

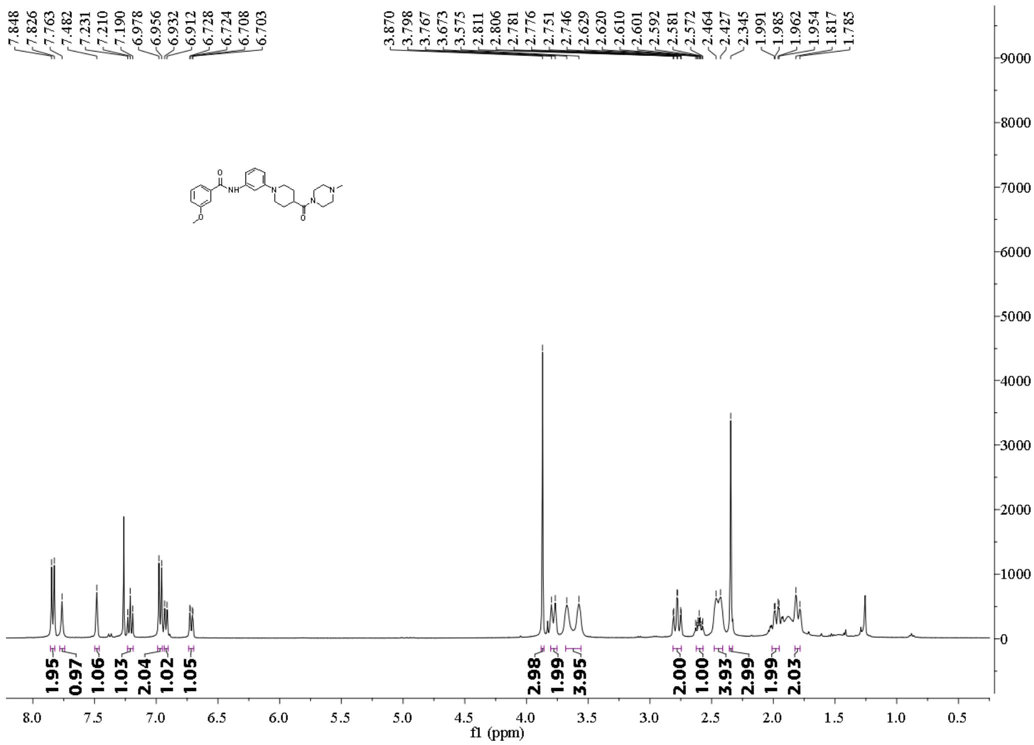

Fig.14-2 ^{1}H-NMR spectrum of compound **5j**

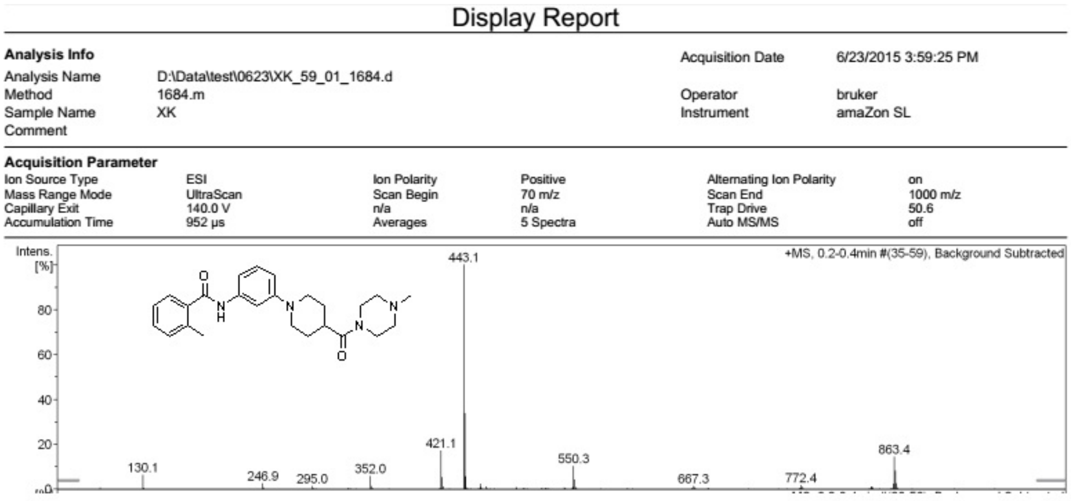

Fig.15-1 MS spectrum of compound **5k**

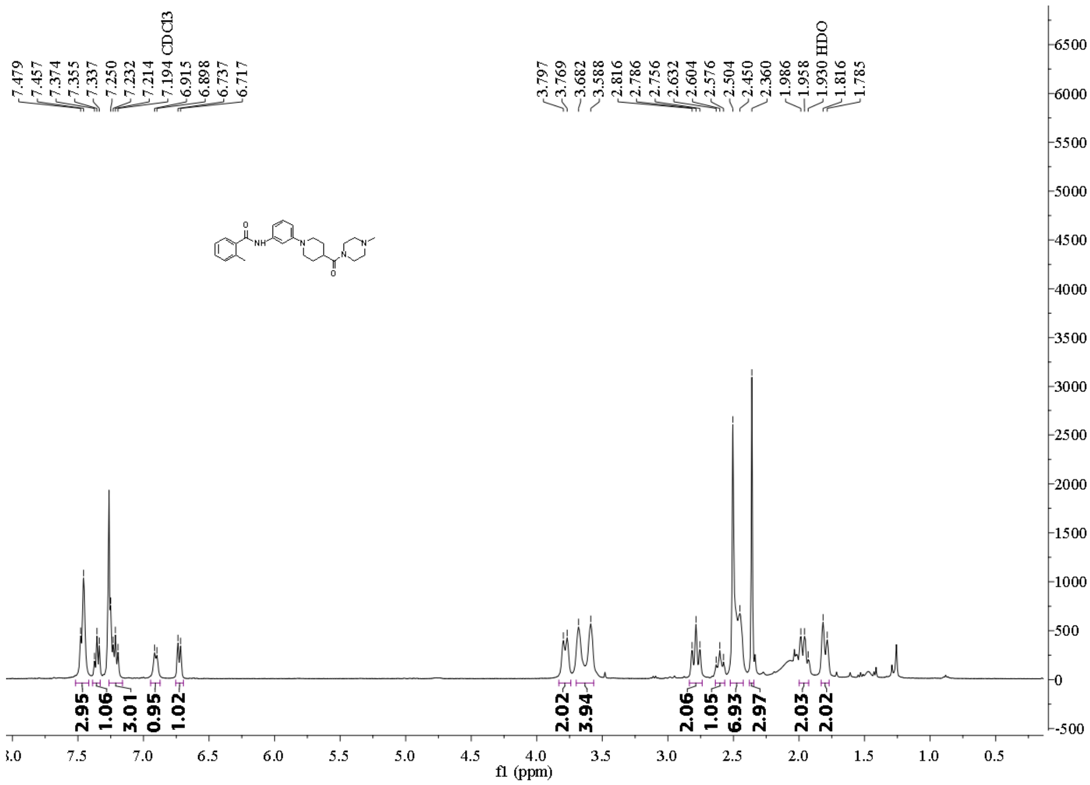

Fig.15-2 ^{1}H-NMR spectrum of compound **5k**

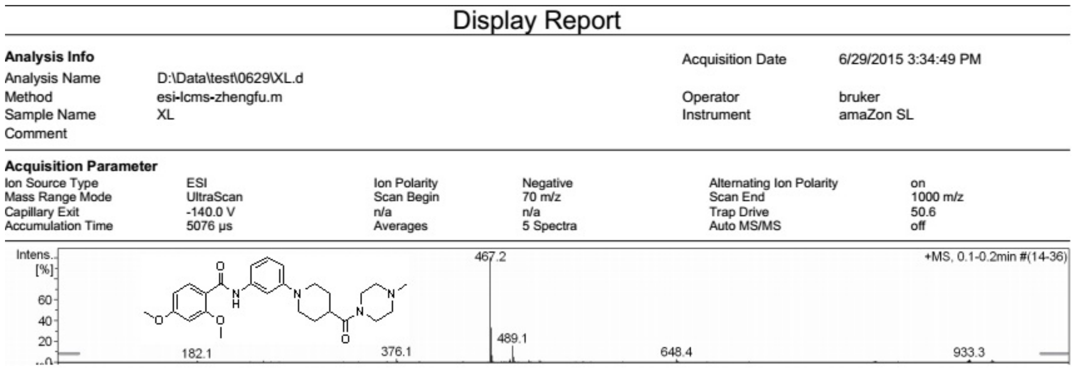

Fig.16-1 MS spectrum of compound **5l**

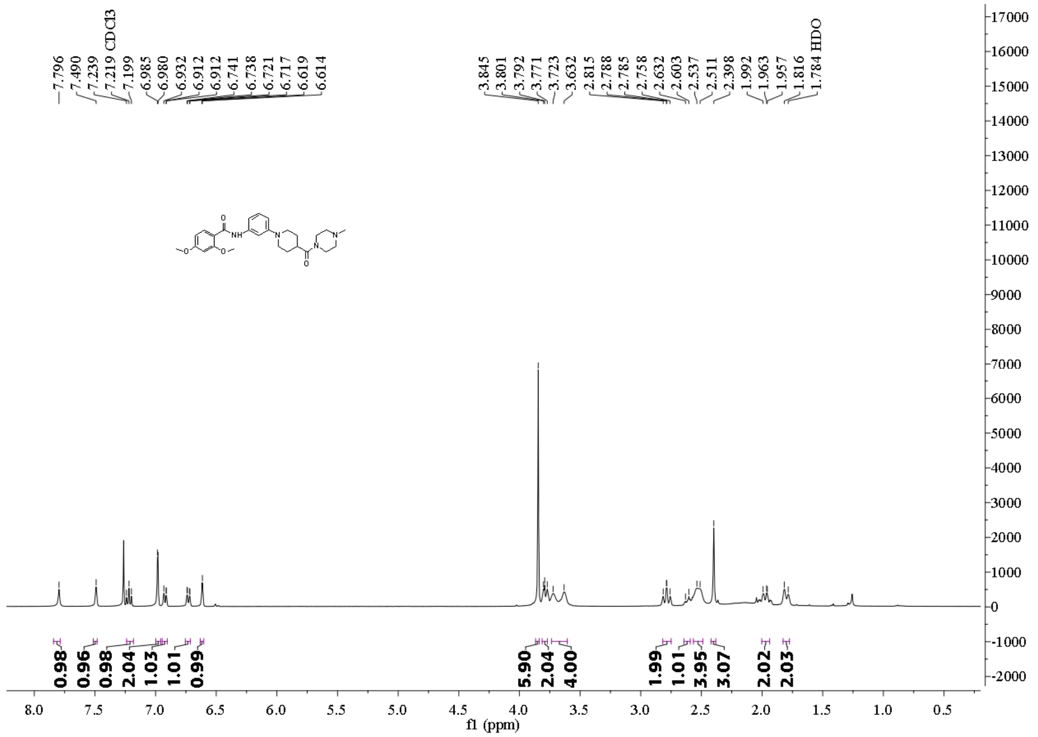

Fig.16-2 ^{1}H-NMR spectrum of compound **5l**

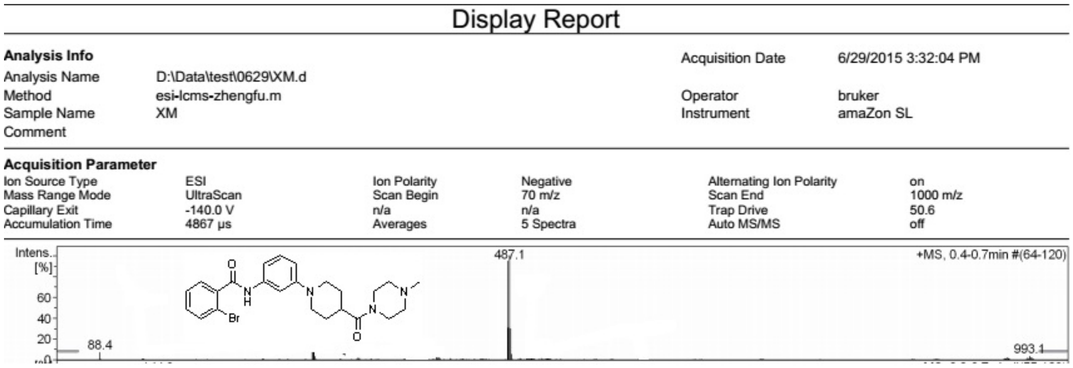

Fig.17-1 MS spectrum of compound **5m**

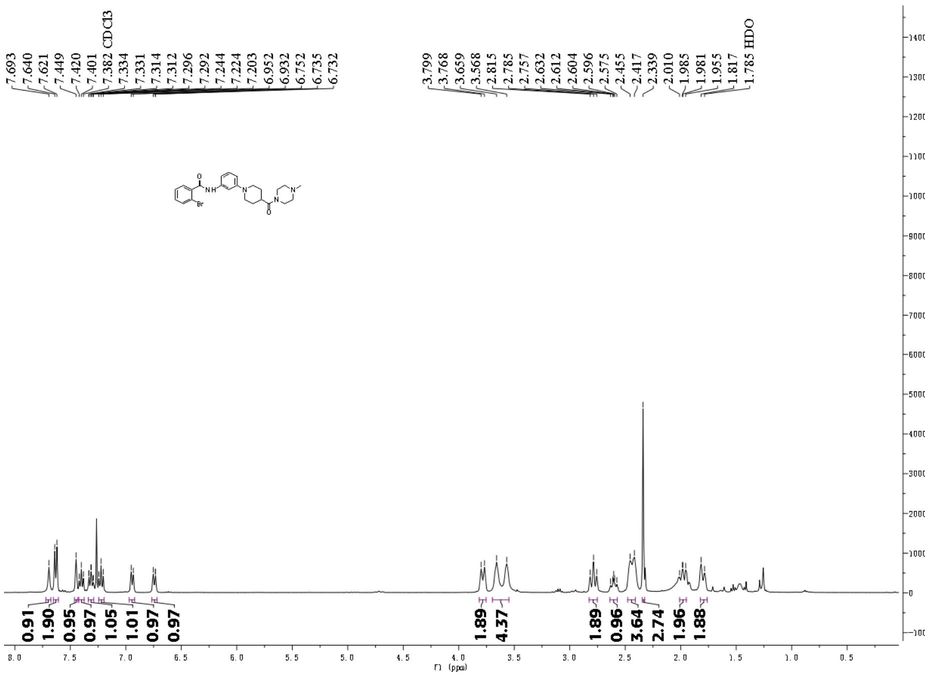

Fig.17-2 ^{1}H-NMR spectrum of compound **5m**

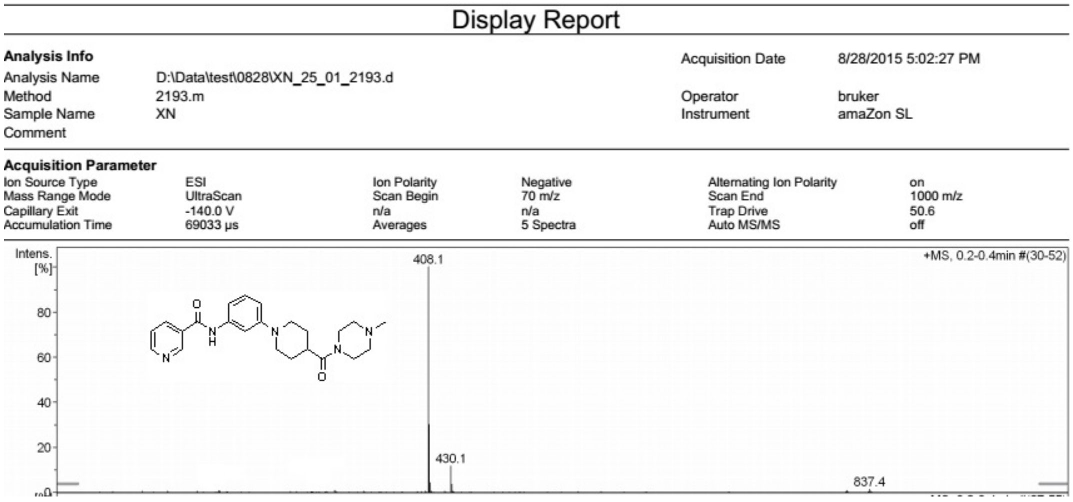

Fig.18-1 MS spectrum of compound **5n**

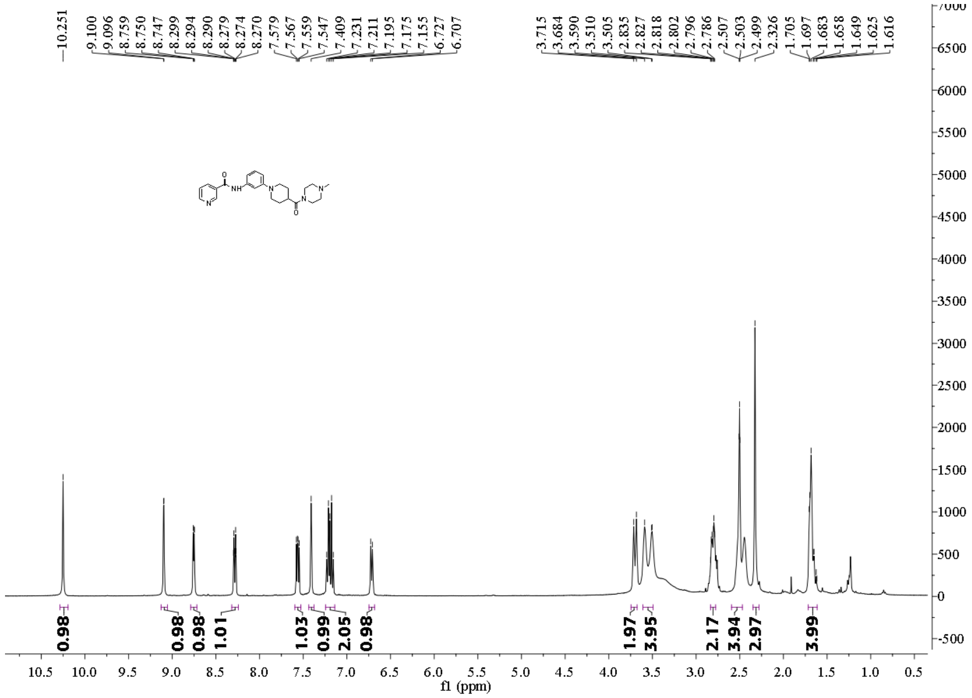

Fig.18-2 ^{1}H-NMR spectrum of compound **5n**

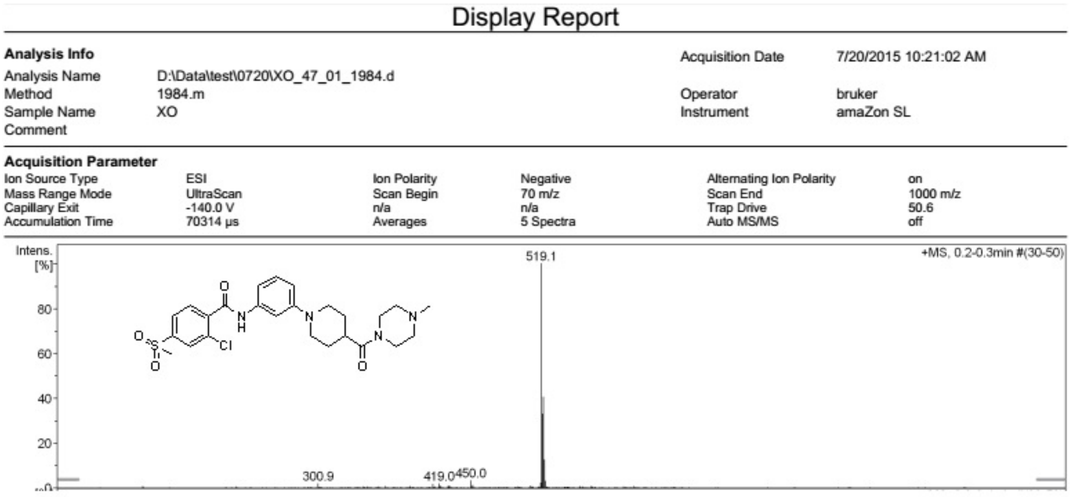

Fig.19-1 MS spectrum of compound **5o**

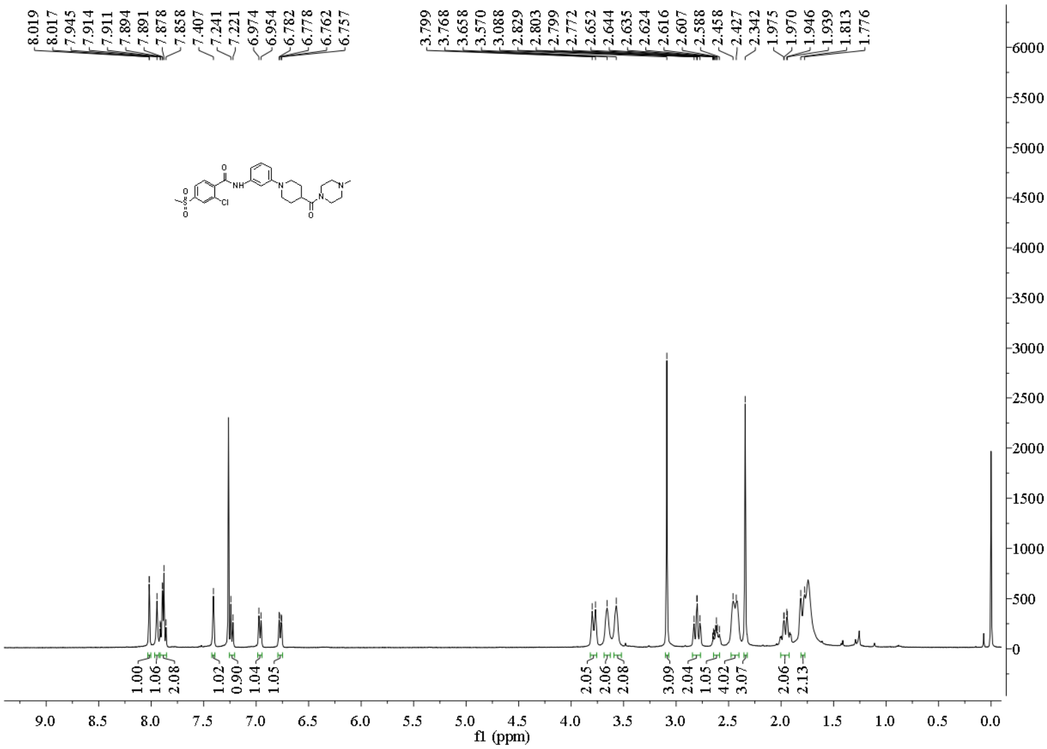

Fig.19-2 ^{1}H-NMR spectrum of compound **5o**

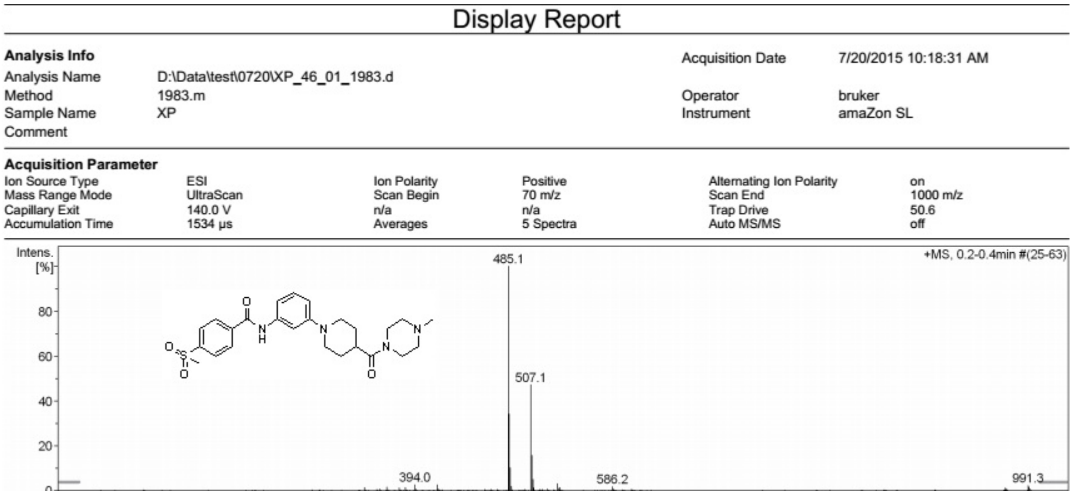

Fig.20-1 MS spectrum of compound **5p**

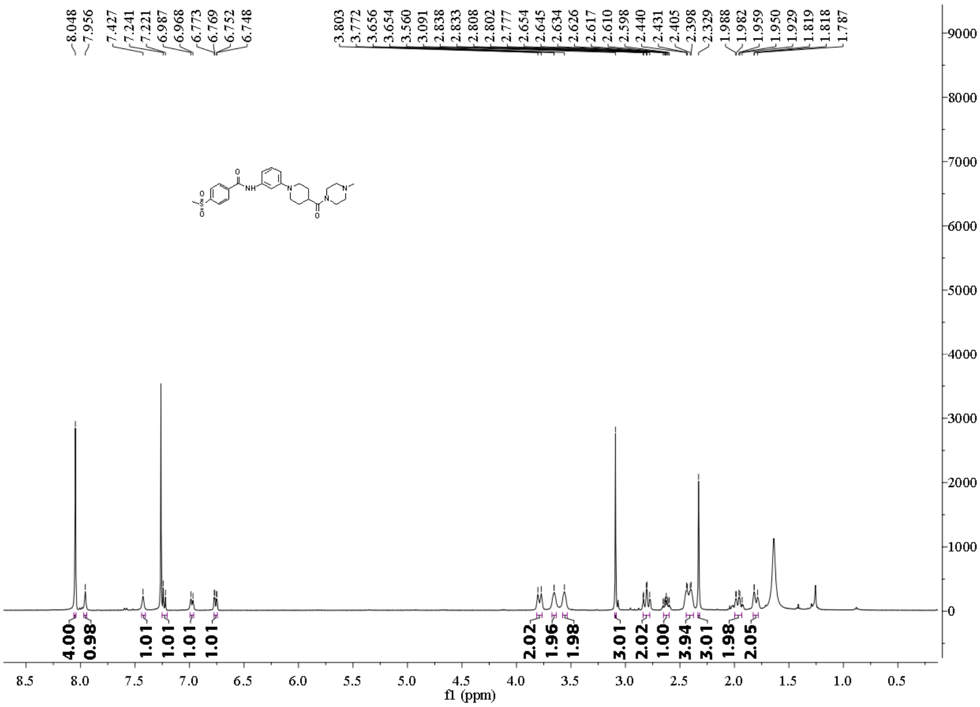

Fig.20-2 ^{1}H-NMR spectrum of compound **5p**

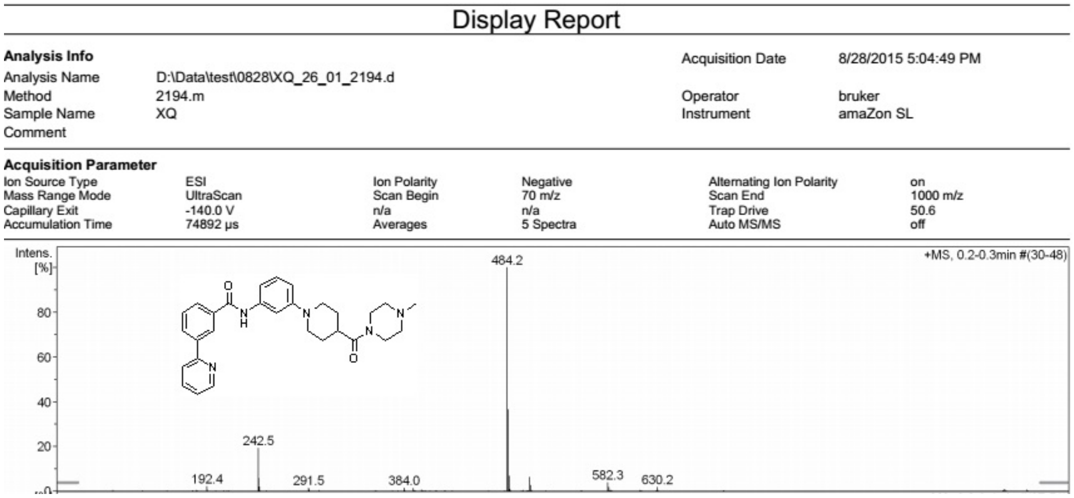

Fig.21-1 MS spectrum of compound **5q**

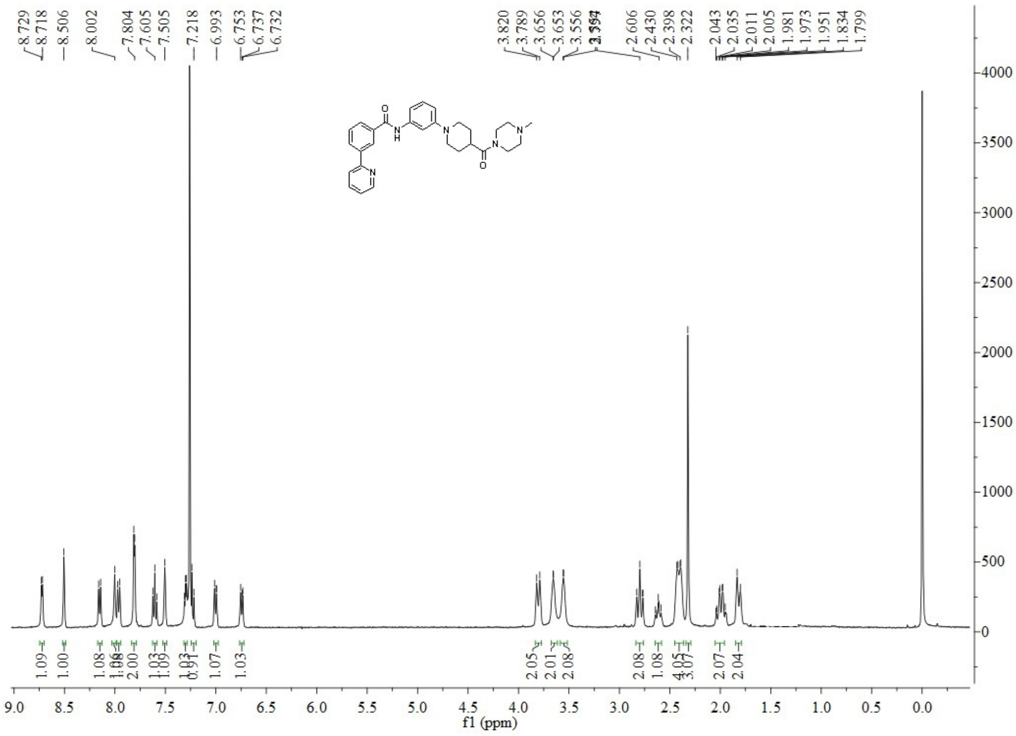

Fig.21-2 ^{1}H-NMR spectrum of compound **5q**

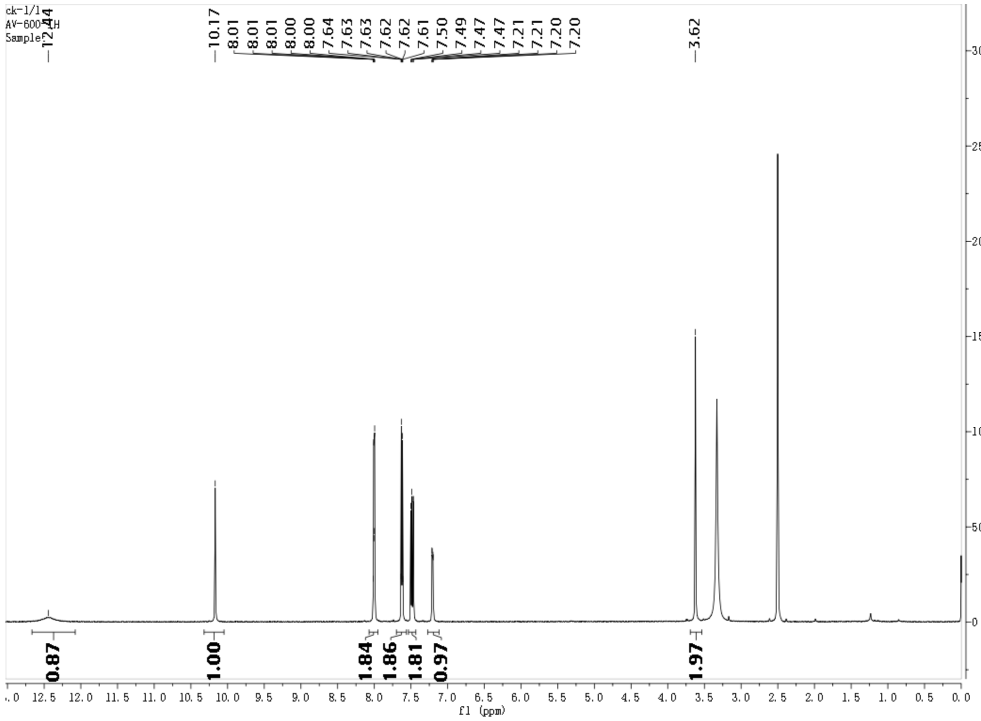

Fig.22 ^{1}H-NMR spectrum of compound **6**

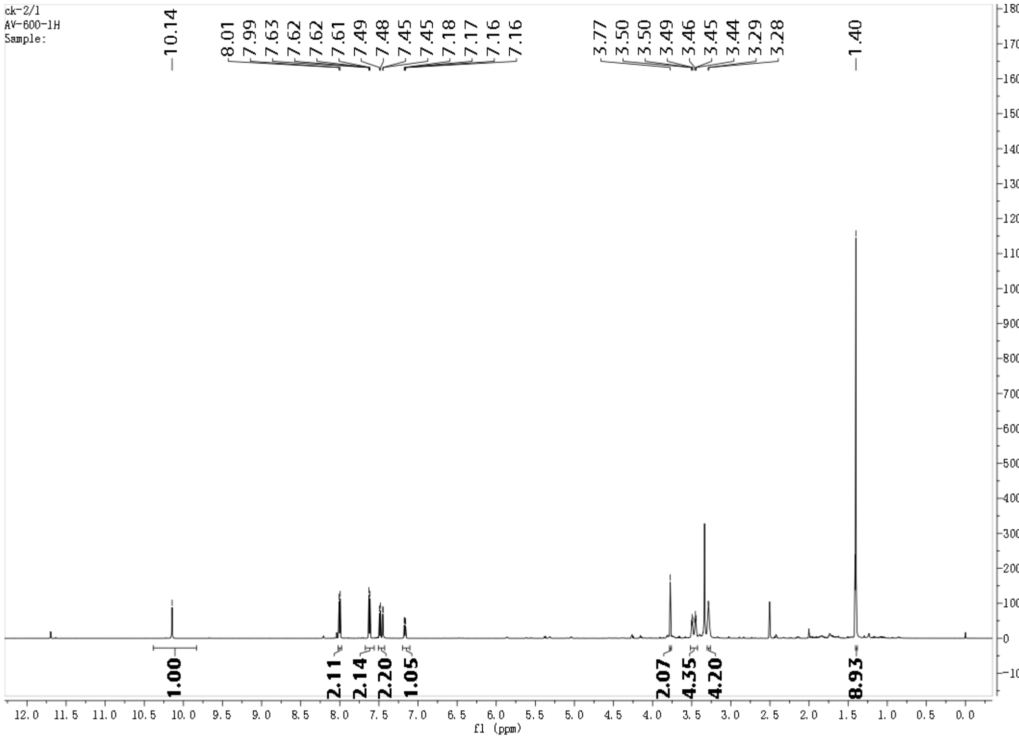

Fig.23 ^{1}H-NMR spectrum of compound **7**

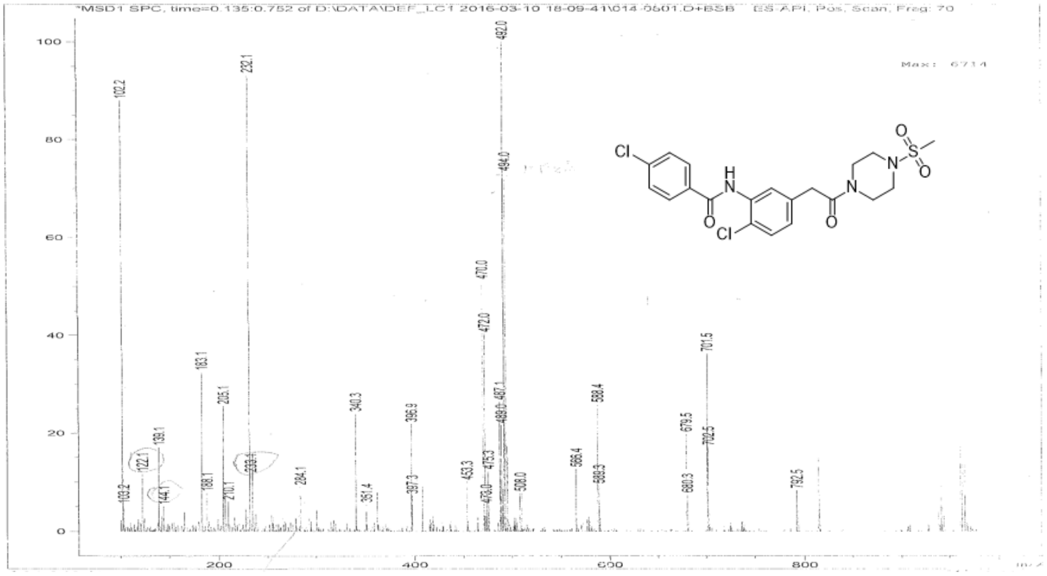

Fig.24-1 MS spectrum of of compound **8a**

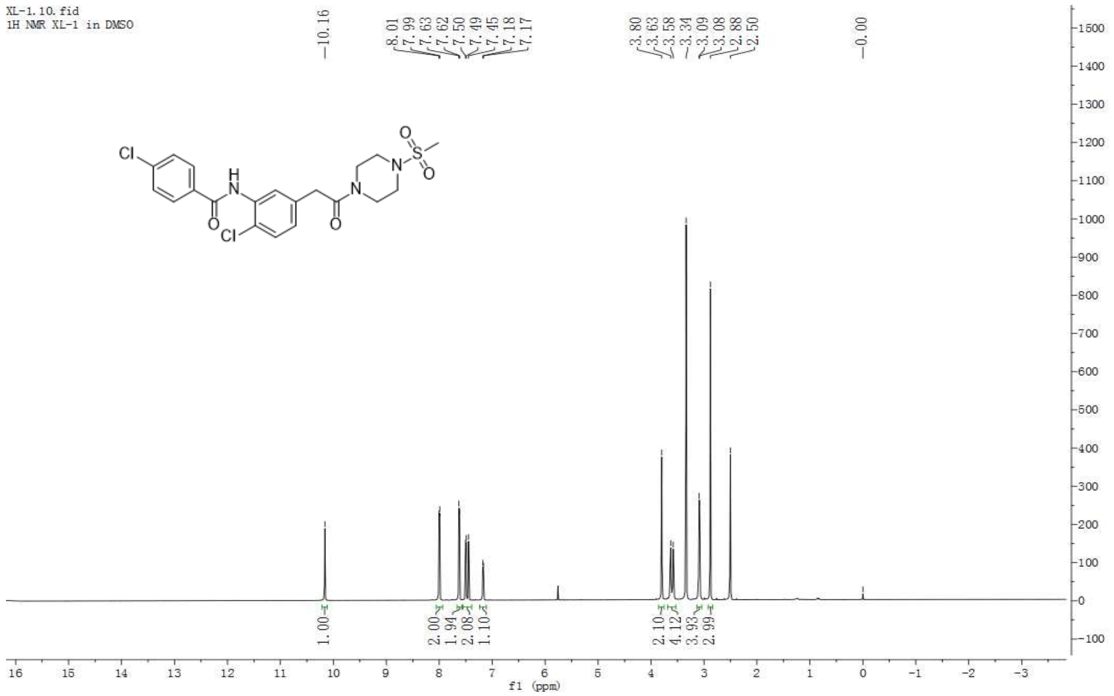

Fig.24-2 ^{1}H-NMR spectrum of compound **8a**

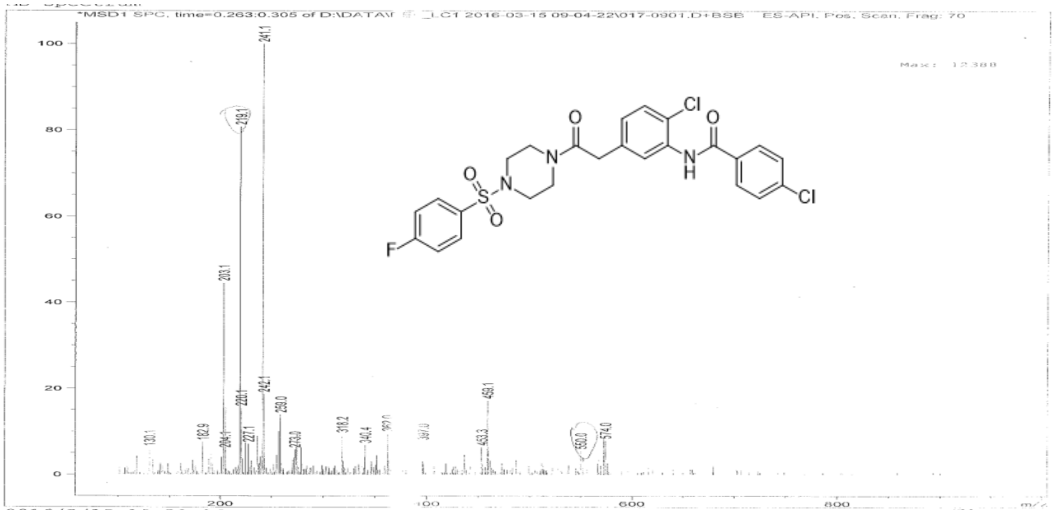

Fig.25-1 MS spectrum of of compound **8b**

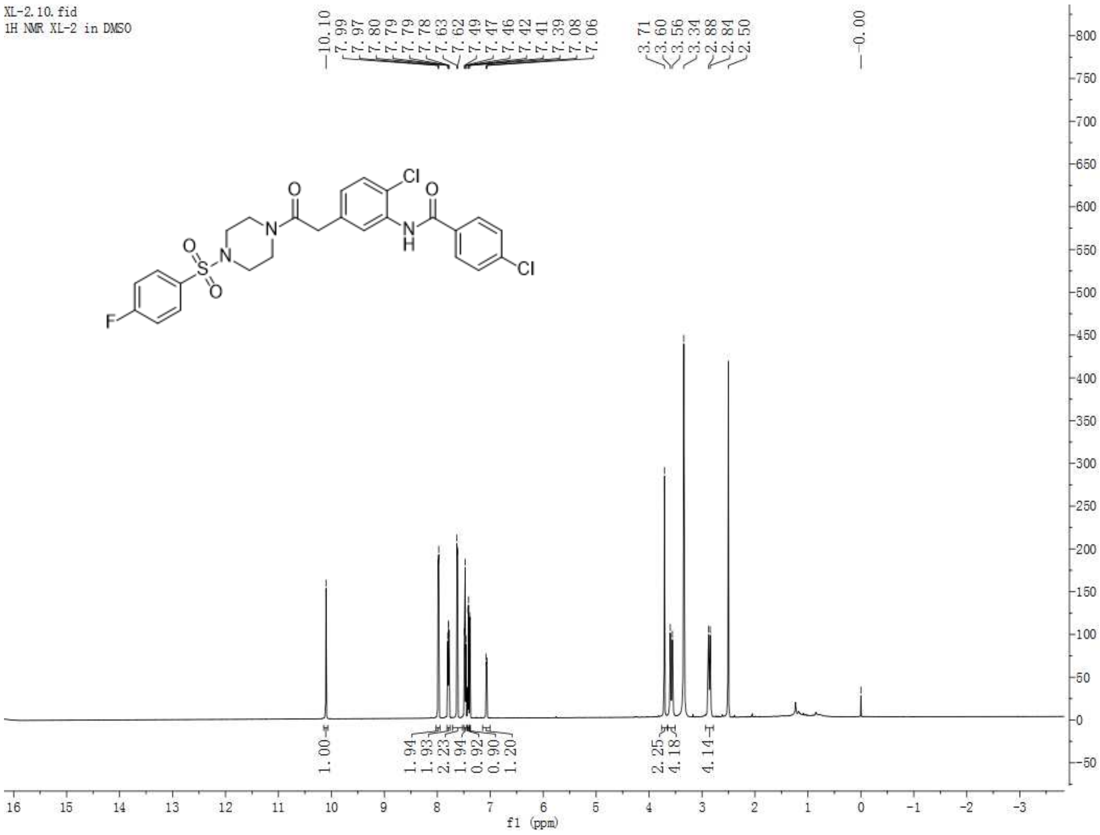

Fig.25-2 ^{1}H-NMR spectrum of compound **8b**

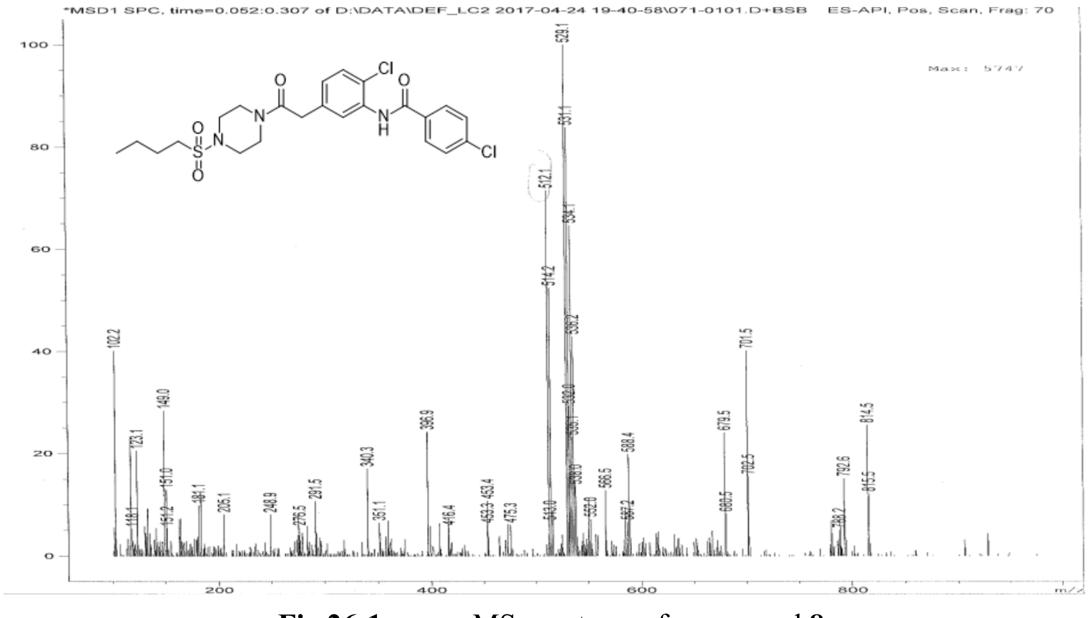

Fig.26-1 MS spectrum of compound **8c**

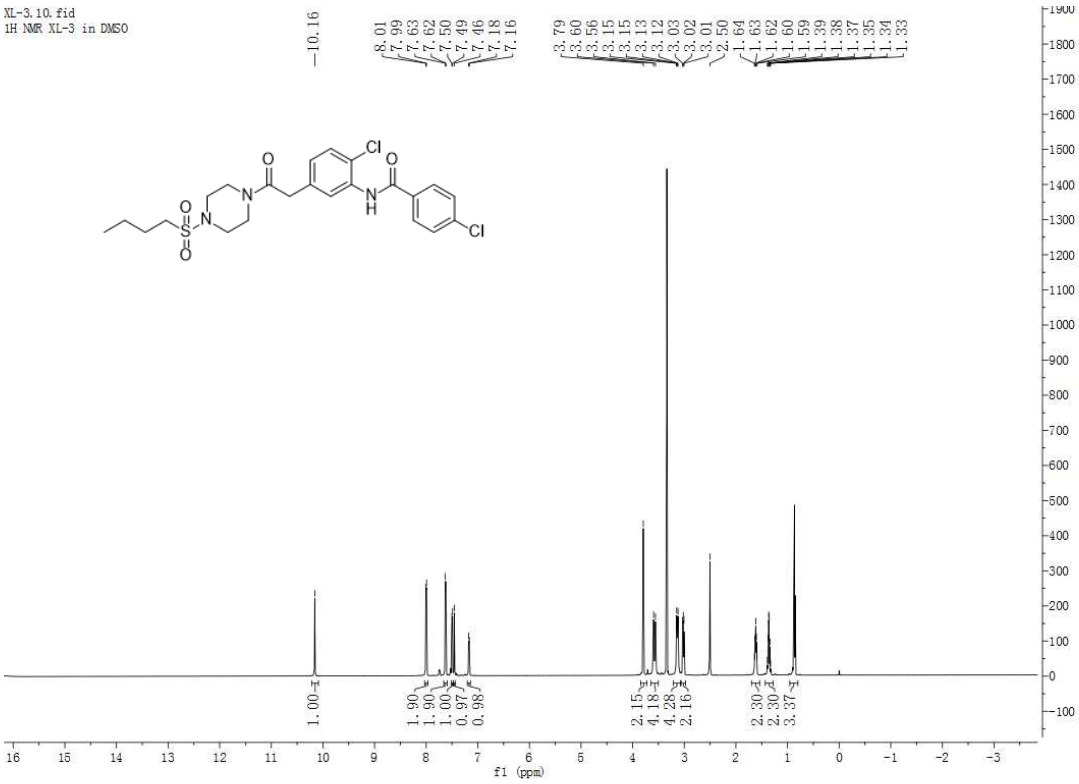

Fig.26-2 ^{1}H-NMR spectrum of compound **8c**
